# Discovery of naturally occurring *ESR1* mutations in breast cancer cell lines modelling endocrine resistance

**DOI:** 10.1038/s41467-017-01864-y

**Published:** 2017-11-30

**Authors:** Lesley-Ann Martin, Ricardo Ribas, Nikiana Simigdala, Eugene Schuster, Sunil Pancholi, Tencho Tenev, Pascal Gellert, Laki Buluwela, Alison Harrod, Allan Thornhill, Joanna Nikitorowicz-Buniak, Amandeep Bhamra, Marc-Olivier Turgeon, George Poulogiannis, Qiong Gao, Vera Martins, Margaret Hills, Isaac Garcia-Murillas, Charlotte Fribbens, Neill Patani, Zheqi Li, Matthew J. Sikora, Nicholas Turner, Wilbert Zwart, Steffi Oesterreich, Jason Carroll, Simak Ali, Mitch Dowsett

**Affiliations:** 1 Breast Cancer Now Toby Robins Research Centre, Institute of Cancer Research, London, SW7 3RP UK; 2 Division of Cancer, CRUK Labs, University of London Imperial College, London, W12 0NN UK; 3 Centre for Cancer Imaging, Institute of Cancer Research, Sutton, SM2 5NG UK; 4 Proteomic Unit, Institute of Cancer Research, London, SW7 3RP UK; 5 Division of Cancer Biology, The Institute of Cancer Research, London, SW3 6JB UK; 6 Division of Computational and Systems Medicine, Department of Surgery and Cancer, Imperial College London, London, SW7 2AZ UK; 7 Ralph Lauren Centre for Breast Cancer Research, Royal Marsden Hospital, London, SW3 6JB UK; 8 Department of Pharmacology and Chemical biology, University of Pittsburgh, Pittsburgh, PA 15213 USA; 9 Department of Molecular Pathology, Netherlands Cancer Institute, 1066CX Amsterdam, Netherlands; 10 Cancer Research UK Cambridge Institute, University of Cambridge, Cambridge, CB2 0RE UK

## Abstract

Resistance to endocrine therapy remains a major clinical problem in breast cancer. Genetic studies highlight the potential role of estrogen receptor-α (*ESR1*) mutations, which show increased prevalence in the metastatic, endocrine-resistant setting. No naturally occurring *ESR1* mutations have been reported in in vitro models of BC either before or after the acquisition of endocrine resistance making functional consequences difficult to study. We report the first discovery of naturally occurring *ESR1* ^*Y537C*^ and *ESR1* ^*Y537S*^ mutations in MCF7 and SUM44 ESR1-positive cell lines after acquisition of resistance to long-term-estrogen-deprivation (LTED) and subsequent resistance to fulvestrant (ICIR). Mutations were enriched with time, impacted on ESR1 binding to the genome and altered the ESR1 interactome. The results highlight the importance and functional consequence of these mutations and provide an important resource for studying endocrine resistance.

## Abstract (web summary)

*ESR1* mutations occur in endocrine-resistant patients but have not yet been reported in in vitro models of breast cancer. Here, the authors report the discovery of naturally occurring *ESR1* ^Y537C^and *ESR1* ^Y537S^ mutations in two breast cancer cell lines after acquisition of resistance to long-term-estrogen-deprivation.

## Introduction

Over 70% of breast cancers (BC) are estrogen receptor-α (ESR1) positive at diagnosis. Estrogen mediates its effects by binding to ESR1 leading to expression of genes controlling proliferation and cell survival. ESR1 has two distinct activation domains, AF-1 and AF-2. AF-1 is regulated by phosphorylation while AF-2 is integral to the ligand-binding domain (LBD) and associates with coactivators, controlling the ESR1 transcriptional complex (reviewed by Green and Carroll^1^). Classically, patients with ESR1-positive BC are treated with endocrine agents such as tamoxifen, aromatase inhibitors (AIs), or fulvestrant, which impede ESR1-signaling (reviewed by Ma et al.^2^). Although over 50% of ESR1-positive patients show response to endocrine therapy and estrogen deprivation therapy reduces BC mortality by 40%^3^, a large proportion relapse with de novo or acquired resistant disease, making it one of the greatest challenges for BC research and treatment.

Multiple mechanisms of resistance have been proposed, most of which have been identified using a limited number of ESR1-positive BC cell lines. These include aberrant cross-talk between ESR1 and growth factor signaling pathways or alterations in the balance of coactivators and corepressors (reviewed by Ma et al.^2^, Osborne et al.^4^, and Miller et al.^5^).

It has been known for many years that some mutations in *ESR1* can lead to ligand-independent activation, but until recently, such mutations appeared to have little clinical significance^6^, as their presence in primary disease is rare. However, the prevalence of *ESR1* mutations in metastatic tumors that have recurred or progressed after endocrine therapy is far higher^7–9^. We have recently reported that the detection of these mutations in circulating tumor DNA (ctDNA) of 39.1% of metastatic patients appears to correlate with clinical resistance to AIs^10^. The majority of *ESR1* mutations are located at two amino acids in the LBD Y537N/C/S and D538G. Functional studies using ectopic expression of these mutations led to constitutive activity of ESR1 and conferred partial resistance to established clinical doses of tamoxifen and fulvestrant^11,12^. However, as these mutations were engineered, the role of cellular context during acquisition of resistance with time was not explored.

In this manuscript, we report for the first time, the identification of naturally occurring *ESR1* mutations in BC cell models and their enrichment during acquisition of resistance to endocrine therapy. We show that the mutated ESR1 controls a cistrome similar to the ligand-dependent wt ESR1 and associates with an altered protein interactome enabling ligand-independent proliferation. Furthermore, these naturally occurring *ESR1* mutants are sensitive to fulvestrant, suggesting that this and similar agents may have applicability in patients with tumors harboring these mutations supporting our recent clinical data^13^.

## Results

### Discovery of *ESR1* mutations in models of endocrine resistance

Previously, we reported the development of long-term-estrogen-deprived (LTED) derivatives from a number of ESR1-positive BC cell lines (including MCF7, HCC1428, T47D, ZR75.1, and SUM44)^14,15^. In general, estrogen deprivation leads to an initial quiescent population accompanied by cell death and after many weeks to outgrowth of a cell population that then proliferates independently of exogenous estrogen (Supplementary Fig. 1a–d). The phenotype of the LTED cell lines varies leading to a context-specific sensitivity or resistance to additional agents^14^.

**Fig. 1:**
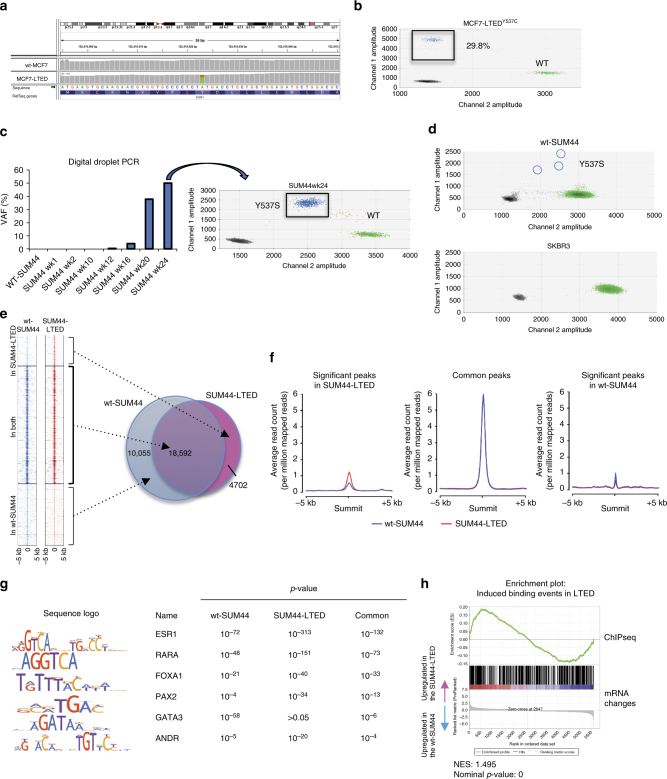
Identification and characterization of *ESR1* mutations in models of endocrine resistance. **a** Visualization of *ESR1* ^*Y537C*^ identified during exome sequencing. **b** Digital droplet PCR (ddPCR) showing the presence of the *ESR1* ^*Y537C*^ mutation in MCF7-LTED. **c** ddPCR showing the presence of the *ESR1* ^*Y537S*^ mutation in SUM44-LTED. Temporal analysis showing enrichment of the mutation from wk12 post-estrogen deprivation. **d** ddPCR showing the presence of *ESR1* ^*Y537S*^ at low variant allele frequency (VAF) in wt-SUM44 but not in SKBR3. **e** Overlap between wt-SUM44 and SUM44-LTED ESR1 binding sites and corresponding heatmap. The heatmap depicts binding peak intensities, which are common or different between the two cell lines. The window represents ±5 kb regions from the center of the binding event. **f** Comparison of the average read count between wt-SUM44 and SUM44-LTED showing peak affinity for the common and different binding events between the two cell lines. **g** Motif analysis of common and augmented ESR1 peaks from wt-SUM44 vs. SUM44-LTED. *p*-value of “common peaks” based on average of three random selections of 2150 peaks to approximately match the number of peaks within the “augmented peak” comparisons. **h** GSEA was conducted comparing RNA-seq with ESR1-induced binding events in SUM44-LTED. ChIP-seq analysis was carried out using data from two biological replicates and RNA-seq from three biological replicates

As *ESR1* mutations have been associated with resistance to endocrine therapy, we explored whether these mutations or those of other genes were either enriched or acquired in the in vitro models described. Whole-exome sequencing from wt-MCF7 and MCF7-LTED showed an *ESR1* ^Y537C^ mutation in the MCF7-LTED at an estimated variant allele frequency (VAF) of 30%, while it was undetectable in the wt-MCF7. The mutation was validated using digital droplet (dd) PCR (Fig. 1a, b).

### *ESR1* mutations occur in LTED but not tamoxifen-resistant cells

As a result of this unexpected finding, we sequenced known hotspot regions for *ESR1* ^16^ by Ion Torrent in wt and LTED derivatives of MCF7, SUM44, HCC1428, and ZR75.1, together with tamoxifen-resistant (TAMR) derivatives of MCF7 and HCC1428 and fulvestrant-resistant (ICIR) derivatives of wt-MCF7, MCF7-LTED, and ZR75.1-LTED (Table 1; Supplementary Fig. 2). The *ESR1* ^*Y537C*^ mutant was detected in the MCF7-LTED-ICIR cells at a VAF of 48% that was confirmed by ddPCR (49.8%) (Supplementary Fig. 3a) but was not detected in the wt-MCF7-ICIR cells. Comparison of the two isogenic models showed that fulvestrant resistance (Supplementary Fig. 3b) occurred irrespective of the mutation. Furthermore, both ICIR derivatives showed a marked reduction in ESR1 (Supplementary Fig. 3c) and a concomitant drop in expression of estrogen-regulated genes (*GREB1, PDZK1, PGR,* and *TFF1*) but equivalent expression of genes associated with proliferation when compared to their respective wt (Supplementary Fig. 3d).

**Table 1:** Identification of naturally occurring *ESR1* mutations in cell line models of endocrine sensitive and resistant breast cancer

Cell line	No. of batches screened	No. of positive batches	Mutation	VAF (%)
wt-MCF7	4	0	–	–
MCF7-LTED	4	1	Y537C	30
MCF7-TAMR	1	0	–	–
wt-MCF7-ICIR	1	0	–	–
MCF7-LTED-ICIR	1	1	Y537C	50
wt-HCC1428	1	0	–	–
HCC1428-LTED	1	0	–	–
HCC1428-TAMR	1	0	–	–
wt-SUM44	2^*^	1	Y537S	0.0001
SUM44-LTED	2^*^	1	Y537S	50
wt-ZR75.1	1	0	–	–
ZR75.1-LTED	1	0	–	–

^*^Second batch originated from an independent laboratory

Strikingly, analysis by Ion torrent also revealed an *ESR1* ^*Y537S*^ heterozygous mutation in SUM44-LTED (VAF 47%). *ESR1* mutations were confirmed by Sanger sequencing, RNA sequencing, mass spectrometry, and whole-exome sequencing (Supplementary Fig. 4a–g). Exome sequencing did not reveal any additional mutated genes involved in AI resistance beyond the mutation in *ESR1* nor did it reveal mutations in genes known to be drivers of BC^17^ that might promote growth by other mechanisms (Supplementary Data 1).

In order to determine if the *ESR1* ^*Y537C*^ VAF of 30% in the MCF7-LTED cells was indicative of a mixed population of cells harboring either *ESR1* ^*w*t^ or *ESR1* ^*Y537C*^, we assessed *ESR1* copy number by fluorescent in situ hybridization (FISH) and exome sequencing. This revealed an allelic imbalance, which on average identified two or more wt copies of *ESR1* and one mutant copy per cell in the MCF7-LTED, indicating 100% of the cell population harbored the mutation. In contrast, the MCF7-LTED-ICIR cells were enriched for two copies of *ESR1* per cell similar to the SUM44-LTED, accounting for the VAF of 50% again indicating every cell in the given population contained a mutation (Supplementary Fig. 5).

### Temporal enrichment of ESR1 mutations during estrogen deprivation

Analysis by ddPCR over a time course showed that the *ESR1* ^*Y537S*^ mutation was detectable within 12 weeks following transfer of SUM44 cells to estrogen-free medium (Fig. 1c). Thereafter, the VAF increased progressively up to 50%. In order to determine if the mutation was present in the parental population or was acquired as a result of the selective pressure of estrogen withdrawal, we screened over 6 × 10^6^ matched parental SUM44 copies. Interestingly, the *ESR1* ^*Y537S*^ mutation was present in wt-SUM44 at an apparent frequency of ~1:1.000.000 (Fig. 1d), implying that the *ESR1* ^*Y537S*^ mutation pre-exists in a very small proportion of SUM44 cells. We further screened a second batch of SUM44-LTED and their corresponding parent cell line^18^ but no mutation was identified, suggesting this is not the only adaptive mechanism. In order to control further the potential of contamination, we screened an equivalent number of ESR1-negative SKBR3 cells and no mutation was evident (Fig. 1d). Finally, to address the possibility that the Y537C mutation was also resident at low frequency in MCF7 cells, we screened three independent batches, covering over 6 × 10^6^ copies, however we were unable to identify the Y537C mutation.

### ESR1^Y537S^ drives ligand-independent transcription

To determine the function of *ESR1* ^*Y537S*^, we performed ChIP-seq with antibodies for ESR1 in asynchronous wt-SUM44 in the presence of estrogen and SUM44-LTED in the absence of estrogen. Overlap of two replicate experiments called 28,647 and 23,294 ESR1 binding events in wt-SUM44 and SUM44-LTED cells, respectively. The vast majority (80%) of the ESR1^Y537S^ binding sites in SUM44-LTED cells were common to ESR1^wt^ binding sites in estrogen-treated wt-SUM44 (Fig. 1e). Although 4702 differential binding sites were called in the SUM44-LTED cells, these were not unique, but represented enriched ESR1 binding, i.e., they also appeared in wt-SUM44 and this was similarly the case for the 10,055 differential binding sites in wt-SUM44 that occurred in the SUM44-LTED but were not enriched to the same level (Fig. 1f).

Peak strength was evaluated at a number of target genes (Supplementary Fig. 6a), where augmented ESR1^Y537S^ binding was evident in SUM44-LTED compared to wt-SUM44. Furthermore, ChIP-qPCR validation assessing recruitment of ESR1^Y537S^ together with FOXA1, a major pioneer factor for ESR1^19^ and CBP required for an authentic ESR1 transcriptional complex^20^, showed enhanced binding to the promoters of *TFF1* and *GREB1* in the SUM44-LTED compared to wt cell line (Supplementary Fig. 6b).

ESR1 binding sites in both cell lines showed a similar pattern of genomic distribution (Supplementary Fig. 6c). Furthermore, the vast majority of binding motifs were similar for ESR1^wt^ and ESR1^Y537S^, however, significant enrichment for motifs representing the transcription factors *ESR1*, *RARA*, *PAX2*, *ANDR*, and *FOXA1* were evident in relation to the enriched ESR1 peaks found in SUM44-LTED, compared to wt-SUM44, which conversely showed increased *GATA3* (Fig. 1g).

**Fig. 2:**
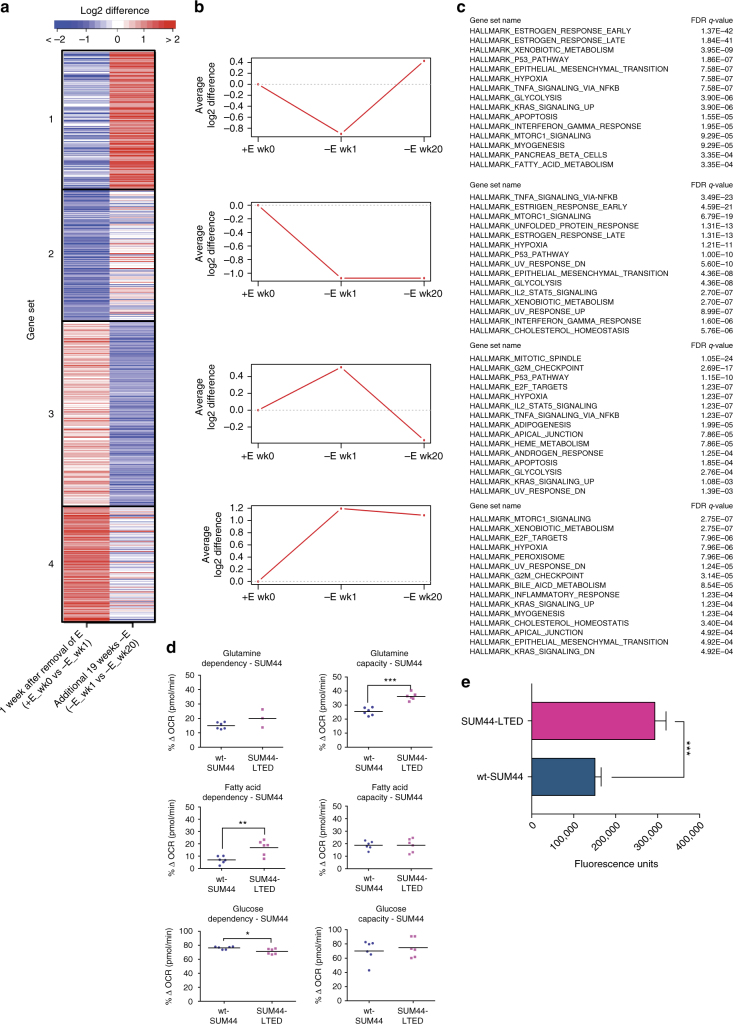
ESR1^Y537S^ controls proliferation, EMT, and altered metabolism in SUM44-LTED. **a** Heatmap depicting the changes in gene expression from four identified clusters of genes that were significantly differentially expressed and bound by ESR1^wt^ (wt-SUM44) or ESR1^Y537S^ (SUM44-LTED). **b** Average log2 differences in ESR1 binding for all genes within each cluster during the course of adaptation to LTED. **c** Pathway analysis of the four clusters using GSEA. Sample labels represent: +E wk0 = wt-SUM44, −E wk1 = 1 wk E-deprived SUM44, −E wk 20 = SUM44-LTED. **d** Metabolic dependency and capacity of wt-SUM44 and SUM44-LTED on glutamine, fatty acid, and glucose using a Seahorse XFe96 analyzer. (*n* = 4 technical replicates). **e** Comparison of the migratory ability of wt-SUM44 and SUM44-LTED (*n* = 8 technical replicates). Error bars represent mean ± SEM. Significance was assessed by Student’s *t* test. *p < 0.05, **p < 0.01, ***p < 0.001

To identify the transcription targets of ESR1^Y537S^, we integrated ChIP-seq and RNA-seq data from the respective cell lines. Gene set enrichment analysis (GSEA) showed that increased *ESR*1^Y537S^ genomic binding correlated with increased transcription, whereas loss of binding correlated with downregulation of genes in SUM44-LTED (Fig. 1h; Supplementary Fig. 6d). We next used K-means clustering to compare the ESR1 binding patterns with expression of genes in wt-SUM44, wt-SUM44 after 1 week of estrogen deprivation and the SUM44-LTED (20 weeks of estrogen deprivation). We identified four distinct gene sets^17^ (Fig. 2a–c): GS1 consisted of classical estrogen-regulated genes such as *TFF1, GREB1, PGR*, and *CCND1*, which decreased in expression after 1 week of deprivation but were elevated in the SUM44-LTED. GS4 contained genes such as *FOXA1* that were enriched after the first week of estrogen deprivation and remained active in the LTED. GS2 and 3 included genes, such as *MYC* and *JUN*, which were downregulated in the SUM44-LTED compared to wt-SUM44. Pathway analysis of the four clusters showed enrichment of ESR1 signaling, epithelial-to-mesenchymal transition (EMT), mTORC1 complex activation, and cholesterol homeostasis in the SUM44-LTED.

To address this further, we assessed the metabolic capability of the wt-SUM44 and SUM44-LTED using Seahorse (Fig. 2d). No significant change in glutamine dependency was evident between the two cell lines; however, the SUM44-LTED showed a significantly higher glutamine capacity and fatty acid dependency compared to the wt-SUM44. The SUM44-LTED also showed a slight but significant decrease in glucose dependency.

Finally, we assessed the migratory ability of the cell lines (Fig. 2e). The SUM44-LTED showed a two-fold increase (*p* < 0.001, Student’s *t* test) in migration compared to wt-SUM44.

Collectively, these findings suggest that *ESR1* ^*Y537S*^ mediates binding events that are functionally significant and lead to expression of genes controlling proliferation, survival and EMT, in a ligand-independent manner and while many ESR1 binding events are similar between the two lines, differences do exist and are probably the result of, or influenced by, the cellular context.

### ESR1^Y537S^ interacts with known ESR1 binding proteins

**Fig. 3:**
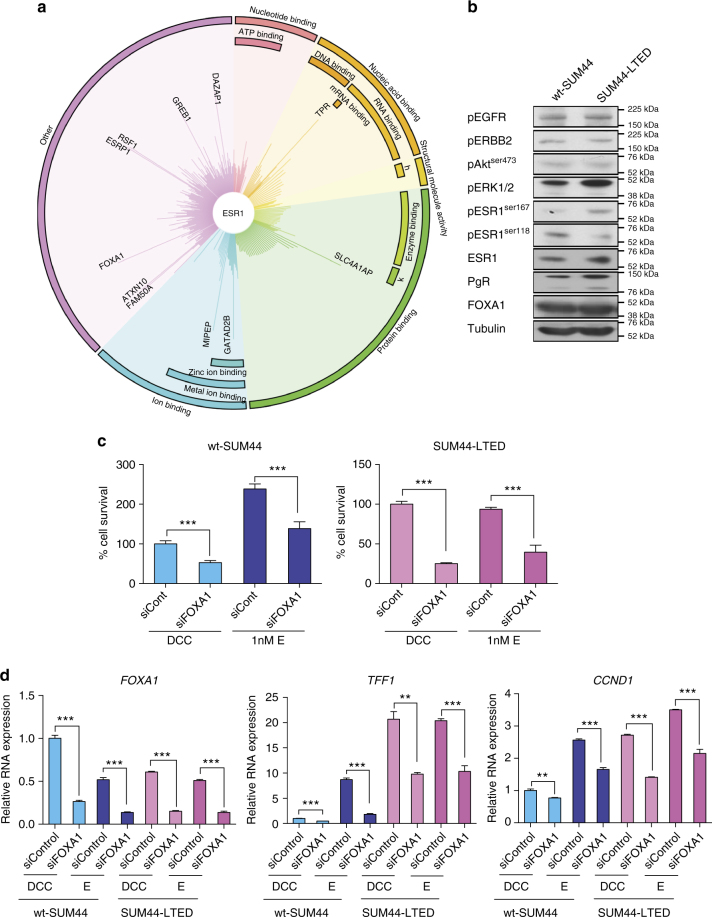
Identification and functional analysis of the ESR1^Y537S^ interactome. **a** MS-ARC depicting ESR1-RIME data conducted on SUM44-LTED (ESR1^Y537S^) vs. wt-SUM44 (ESR1^wt^) (*n* = 3 biological replicates). The ranking is based on SUM44-LTED/wt-SUM44 peptide (razor and unique) counts. The length of the line represents the number of identified peptides. The longer the line, the greater the interaction with ESR1^Y537S^ compared to ESR1^wt^. The shorter cloud of lines shows the high degree of commonality in ESR1 binding proteins between both cell lines. **b** Immunoblotting showing alterations in expression of key protein markers previously associated with endocrine-resistant phenotypes. **c** Proliferation assays following siFOXA1 in wt-SUM44 and SUM44-LTED relative to siControl in the presence and absence of E (estradiol) (*n* = 2 biological experiments with eight technical replicates). **d** Expression of estrogen-regulated genes, *TFF1*, and *CCND1* following suppression of FOXA1 (*n* = 3 technical replicates). (error bars represent mean ± SEM, *p < 0.05, **p < 0.01, ***p < 0.001, significance was assessed by Student’s *t* test)

In order to elucidate the impact of the *Y537S* mutation on the ESR1 interactome and proteome, we carried out comparative RIME (rapid immunoprecipitation with tandem mass spectrometry of endogenous proteins) and dimethyl labeling^21^ between wt-SUM44 and SUM44-LTED (Fig. 3a; Supplementary Fig. 7a, b). RIME demonstrated ESR1^Y537S^ associated with a similar portfolio of proteins to those seen for ESR1^wt^ including ESR1 itself, as well as, PGR, TLE3, HAT1, and FOXA1^22^. However, increased association between ESR1^Y537S^ GREB1 and FOXA1 was noted, which we confirmed by Co-IP (Supplementary Fig. 7c). Quantitation of proteins by dimethyl labeling showed increased abundance of TFF1 and a slight increase in ESR1 but not FOXA1 (Supplementary Fig. 7d).

Immunoblot analysis of wt-SUM44 and SUM44-LTED under basal growth conditions was assessed for changes in growth factor receptors and down stream pathways associated with endocrine resistance^2^ as well as alterations in pESR1^ser118^, pESR1^ser167^, and PGR (Fig. 3b; Supplementary Fig. 8). No significant changes in pEGFR or pERBB2 were apparent between the cell lines. A slight increase in pERK1/2 was seen in SUM44-LTED but no change in pAKT^ser473^. The level of pESR1^ser118^ was greater in wt-SUM44 compared to the SUM44-LTED. However, a slight increase in pESR1^ser167^ was noted in the LTED model (Fig. 3b). To address this further, both wt-SUM44 and SUM44-LTED were cultured in DCC medium in the absence or presence of estrogen. In this setting, ESR1 abundance and phosphorylation profiles were similar between the SUM44-LTED in the absence of estrogen and the wt-SUM44 in the presence of estrogen. Overall, these data showed the profile of the wt-SUM44 and SUM44-LTED were similar (Supplementary Fig. 7e).

As FOXA1 is an important pioneer factor regulating ESR1-driven transcription^23^, and FOXA1 sites were enriched in our ChIP-seq analysis of SUM44-LTED cells, we hypothesized that it played a pivotal role in transcriptional regulation of ESR1^Y537S^. Small interfering RNA (siRNA) knockdown of FOXA1 significantly reduced proliferation of both wt-SUM44 (42%, *p* < 0.001, Student’s *t* test) and SUM44-LTED cells although this was more pronounced in the latter (75%, *p* < 0.001, Student’s *t* test) (Fig. 3c). siFOXA1 also correlated with a significant reduction in the expression of *TFF1* and *CCND1* (Fig. 3d), suggesting FOXA1 plays a crucial role in the ligand-independent transcriptional activity of ESR1^Y537S^.

### CRISPR analysis shows *ESR1*^*Y537S*^ controls ligand independence

**Fig. 4:**
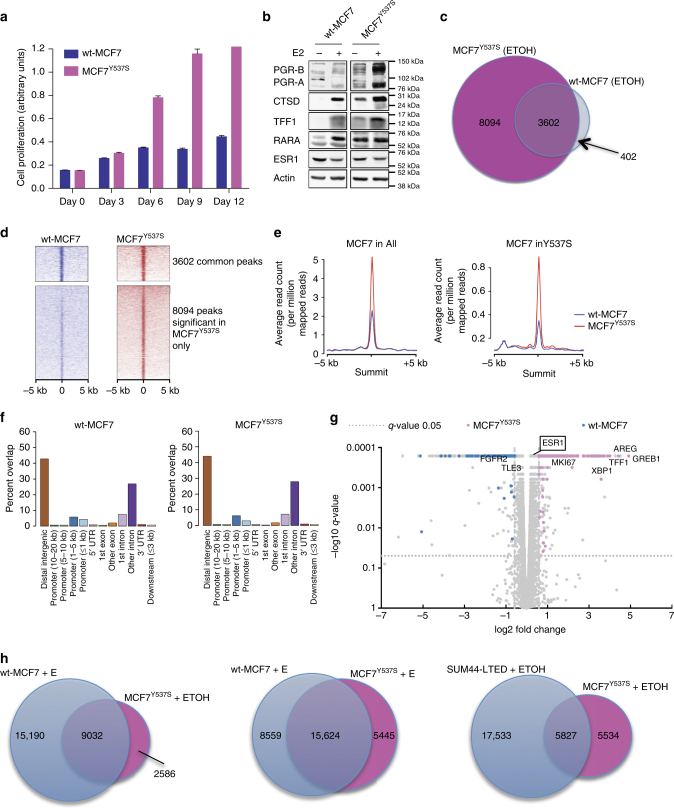
Characterization of CRISPR-cas9-modified wt-MCF7 expressing *ESR1* ^*Y537S*^. **a** Viability assay showing MCF7^Y537S^ proliferates in the absence of exogenous E compared to wt-MCF7 (*n* = 6 technical replicates and three biological replicates). Mean growth at day12 ± SEM relative to day 0. **b** Immunoblotting showing alterations in the expression of ESR1, PGR, CTSD, TFF1, and RARA. **c** Overlap between wt-MCF7 and MCF7^Y537S^ ESR1 binding sites in the absence of E and **d** corresponding heatmap. The heatmap depicts binding peak intensities that are common or different between the wt-MCF7 and MCF7^Y537S^. The window represents ±5 kb regions from the center of the binding event. **e** Comparison of the average read count between wt-MCF7 and MCF7^Y537S^ in the absence of E showing peak affinity in both cell lines (left) and those binding sites only significant in MCF7^Y537S^ (right) (*q*-value < 0.05). **f** Bar chart showing the genomic distribution of ESR1 binding sites across the genome in both cell lines. **g** Volcano plot showing changes in gene expression by RNA-seq as a result of differential ESR1^Y537S^ binding in MCF7^Y537S^ showing increased expression of estrogen-regulated and proliferation-associated genes. **h** Venn-diagrams showing intersect between wt-MCF7 and CRISPR generated MCF7^Y537S^ ChIP-seq peaks in response to ethanol (ETOH) or estradiol (E) and intersect between SUM44-LTED and MCF7^Y537S^ in the absence of E

As kinase signaling has been strongly implicated in endocrine resistance resulting in ligand-independent activity of ESR1^2^, we sought an approach that would reduce the effect of this confounding influence. In this setting, wt-MCF7, which harbor *ESR1* ^*wt*^, were engineered to introduce the *ESR1* ^*Y537S*^ mutation using CRISPR-Cas9 genome editing. MCF7^Y537S^ cells carry one endogenous *ESR1* gene in which, *ESR1* ^*wt*^ has been mutated to code for the *ESR1* ^*Y537S*^ mutation, as well as *ESR1* ^*wt*^. Detailed functional analyses of MCF7^Y537S^ cells are described elsewhere^24^. Proliferation assays in the absence of exogenous estrogen showed the MCF7^Y537S^ was ligand-independent (Fig. 4a). Furthermore, levels of ESR1 expression between the wt and the mutated cell line were similar (Fig. 4b; Supplementary Fig. 8). Analysis of ESR1 ChIP-seq from wt-MCF7 and MCF7^Y537S^ in the absence of exogenous estrogen showed 3602 common peaks across the genome and 8094 unique binding events in MCF7^Y537S^ (Fig. 4c, d). Furthermore, peak affinity was greater for ESR1^Y537S^ across the genome while binding events were similarly distributed for both ESR1^wt^ and ESR1^Y537S^ (Fig. 4e, f). Overlay of the binding events from ChIP-seq analysis with corresponding RNA-seq from MCF7^Y537S^ showed increased expression of proliferation-associated genes and known estrogen-regulated genes, which was confirmed by protein expression (Fig. 4b, g; Supplementary Fig. 8). This data suggest the mutation alone is sufficient to hold ESR1 in a conformation suitable for recruitment of coactivators together with the basal transcription machinery and that these mutations may not require altered kinase profiles to be active. Of note, treatment of both cell lines with estrogen revealed 74% concordance in ESR1 binding events suggesting ESR1^Y537S^ remained responsive to ligand (Fig. 4h).

Intersect of the ESR1 binding events in SUM44-LTED^Y537S^ and MCF7^Y537S^ (Fig. 4h) showed over 50% of the peaks called in MCF7^Y537S^ were common to those in SUM44-LTED^Y537S^. Overlay of the common binding events with RNA-seq showed enrichment of genes associated with Hallmark pathways such as early (*p*-value = 10^−72^, hypergeometric test) and late (*p*-value = 10^−43^, hypergeometric test) estrogen responsiveness, EMT transition (*p*-value = 10^−18^, hypergeometric test), mTORC1 signaling (*p*-value = 10^−12^, hypergeometric test), and fatty acid metabolism (*p*-value = 10^−9^, hypergeometric test) (Supplementary Data 2). Nonetheless, differences between the cell lines highlight the influence of phenotypic nuances on the ESR1 function.

### ESR1^wt^ and ESR1^Y537C^ have altered genome-wide binding patterns

**Fig. 5:**
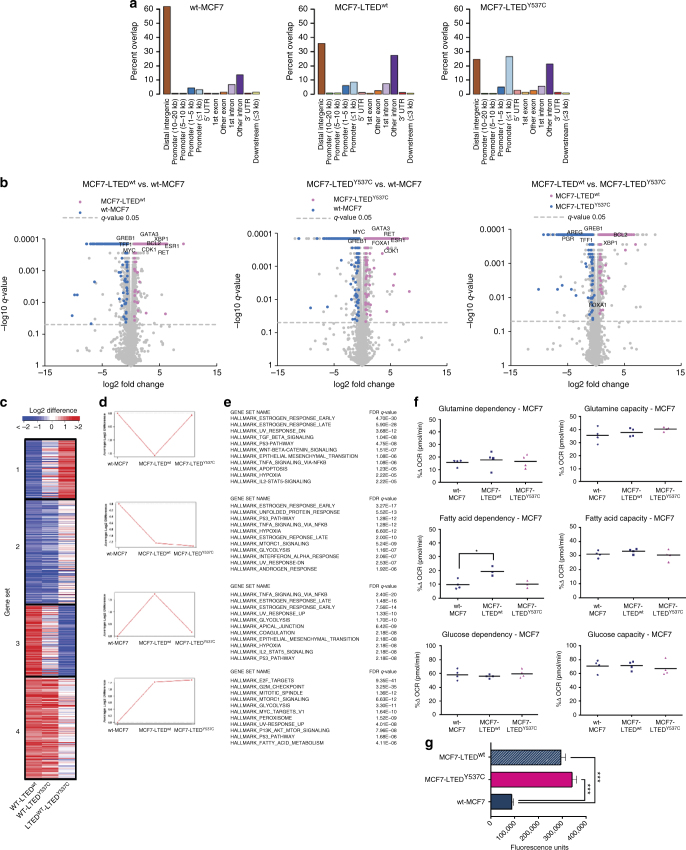
ESR1^wt^ and ESR1^Y537C^ regulate altered ESR1 cistrome. **a** Bar chart showing the genomic distribution of ESR1 binding sites across the genome in wt-MCF7, MCF7-LTED^wt^, and MCF7-LTED^Y537C^ showing altered promoter (≤1 kb) and distal intergenic occupancy. **b** Volcano plots showing changes in gene expression by RNA-seq in MCF7-LTED^Y537C^, MCF7-LTED^wt^, and wt-MCF7. **c** Heatmap depicting the changes in gene expression of the four clusters comparing wt-MCF7 to MCF7-LTED^Y537C^, wt-MCF7 to MCF7-LTED^wt^, and MCF7-LTED^Y537C^ to MCF7-LTED^wt^. **d** Average log2 differences for all genes within each set for wt-MCF7, MCF7-LTED^wt^, and MCF7-LTED^Y537C^. **e** Pathway analysis of the four clusters using GSEA. Data were derived from *n* = 2 biological replicates for ChIP-seq and *n* = 3 biological replicates for RNA-seq. **f** Metabolic dependency and capacity of wt-MCF7, MCF7-LTED^wt^, and MCF7-LTED^Y537C^ on glutamine, fatty acid, and glucose using a Seahorse XFe96 analyzer (*n* = 4 technical replicates). Significance was assessed by one-way ANOVA and Tukey’s test. *p < 0.05, **p < 0.01, ***p < 0.001. **g** Comparison of the migratory ability of wt-MCF7, MCF7-LTED^wt^, and MCF7-LTED^Y537C^ (*n* = 8 technical replicates). Data shown are mean ± SEM. Significance was assessed by Student’s *t* test. *p < 0.05, **p < 0.01, ***p < 0.001

Two MCF7-LTED derivatives were sequenced, of which one harbored an *ESR1* ^*Y537C*^ (MCF7-LTED^Y537C^) and the other *ESR1* ^*wt*^ (MCF7-LTED^wt^) (as confirmed by ddPCR Supplementary Fig. 9a), suggesting LTED itself may not always select for mutations. Indeed, there are no previous reports of *ESR1* mutations in LTED cells. Further interrogation of the whole-exome sequencing data from both MCF7-LTED models showed an increased mutational load in the MCF7-LTED^Y537C^ compared to the MCF7-LTED^wt^. However, no high impact mutations previously associated with AI resistance^2^ were evident in either cell line other than ESR1^Y537C^ (Supplementary Data 1). Immunoblotting showed that while key signaling pathways appeared similar between the LTED derivatives, expression of PGR differed significantly (Supplementary Fig. 9b). We therefore hypothesized that the mutant *ESR1* ^*Y537C*^ and *ESR1* ^*wt*^ controlled different ESR1 cistromes. To address this, genome-wide binding of ESR1 was assessed in both MCF7-LTED derivatives and the corresponding wt-MCF7. Assessment of the distribution of ESR1 binding showed increased occupancy at the promoter (<1kb) in MCF7-LTED^wt^ (9.2%, *p* = 10^−94^ *χ* ^2^-test) and MCF7-LTED^Y537C^ (28.4%, *p* = 0 *χ* ^2^-test) compared to wt-MCF7 (3.3%). The converse was observed for the distal intergenic regions (Fig. 5a). To address this further, we used DiffBind and identified 4744 differential binding events between the MCF7-LTED^wt^ and wt-MCF7, 13,824 between MCF7-LTED^Y537C^ and wt-MCF7, and 11,018 between MCF7-LTED^wt^ and MCF7-LTED^Y537C^ (FDR < 5%) (Supplementary Fig. 9c, d). This suggested that the ESR1^Y537C^ and ESR1^wt^ in the MCF7-LTED cell lines control altered cistromes in comparison to wt-MCF7, but also differed between each other. Of interest, both LTED cell lines showed increased expression of *GATA3, CDK1, RET*, and *ESR1* compared to the parental cell line (Fig. 5b). However, MCF7-LTED^Y537C^ showed increased expression of estrogen-regulated genes such as *PGR* and *TFF1* together with *AREG*, while MCF7-LTED^wt^ showed increased expression of *BCL2* and *XBP1* (Fig. 5b). K-means clustering of the ChIP-seq and RNA-seq data confirmed that the ESR1^Y537C^ mutation appeared to function “classically” in the absence of ligand compared to MCF7-LTED^wt^. Noteworthy, both LTED derivatives enriched for pathways associated with PI3K/AKT/mTORC compared to wt-MCF7 but differed in the down stream impact of these pathways when comparing clusters 1 and 3 (Fig. 5c–e).

We next assessed the metabolic capability of the cell lines, which was similar for both capacity and dependency on glutamine, and glucose (Fig. 5f). However, the MCF7-LTED^wt^ showed higher dependency on fatty acids (*p* < 0.05, one-way ANOVA and Tukey’s test).

Finally, and in keeping with the SUM44-LTED, both MCF7-LTED derivatives were highly migratory compared to wt-MCF7 (Fig. 5g).

In order to further delineate the dependency of the MCF7-LTED^Y537C^ on the mutant *ESR1*, we carried out a CRISPR-Cas9 reversion editing Y537C to Y537 (ESR1^∆537C^) (Supplementary Fig. 10a, b). In keeping with our previous data, MCF7-LTED^Y537C^ showed ligand-independent growth. Contrastingly, MCF7-LTED^∆537C^ and wt-MCF7 revealed limited proliferation in the absence of estrogen (Supplementary Fig. 10c). Furthermore, MCF7-LTED^∆537C^ switched to estrogen dependency and phenocopied the response of wt-MCF7 to fulvestrant (Supplementary Fig. 10d, e). Immunoblotting and RT-qPCR showed that MCF7-LTED^∆537C^ regain estrogen dependency for expression of target genes, *PGR*, *TFF1*, *GREB1*, and *CTSD* (Supplementary Fig. 10f, g). Taken together, these data show that the *ESR1* ^*Y537C*^ mutation is paramount for the ligand-independent phenotype of MCF7-LTED^Y537C^ cells.

### ESR1 mutations show altered responses to endocrine therapy

**Fig. 6:**
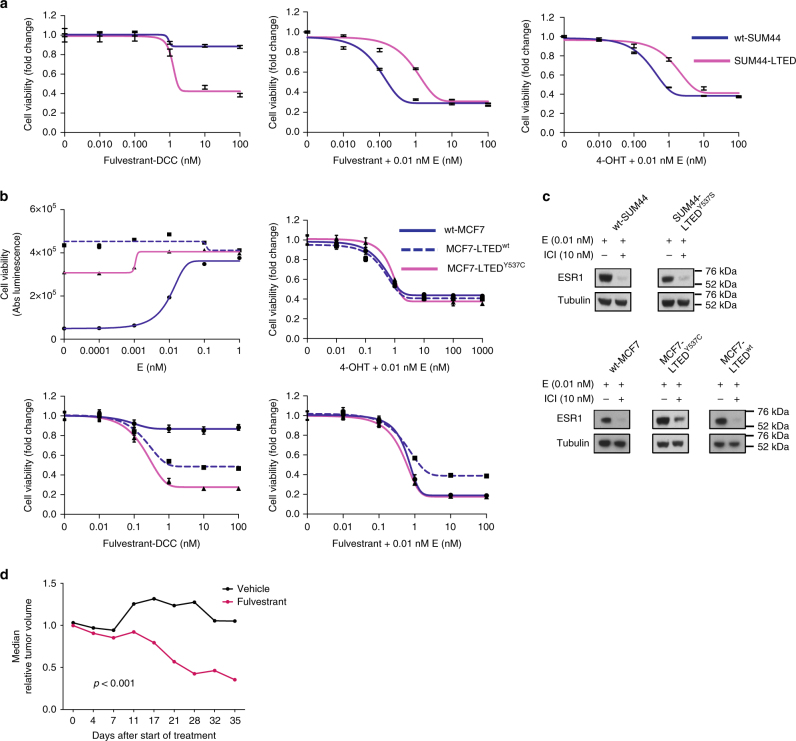
Antiproliferative effect of endocrine therapy in ESR1 mutant and wt cell lines. **a** Proliferation assays assessing response of wt-SUM44 and SUM44-LTED and **b** wt-MCF7, MCF7-LTED^wt^, and MCF7-LTED^Y537C^ to escalating concentration of fulvestrant ± E (estradiol) and 4-OHT plus E (estradiol). **c** Treatment of wt-SUM44, SUM44-LTED^Y537S^, wt-MCF7, MCF7-LTED^Y537C^, and MCF7-LTED^wt^ with fulvestrant (10 nM) results in loss of ESR1 expression irrespective of mutation status (*n* = 3 biological replicates consisting of *n* = 8 technical replicates). Data represent mean ± SEM. **d** Xenograft models of MCF7-LTED^Y537C^ in response to vehicle or fulvestrant. Data represent median fold change in tumor volume. Significance was assessed using an unpaired *t*-test

One of the most clinically pressing questions relates to the sensitivity of ESR1 mutations to endocrine therapy. Cell lines were treated with escalating concentrations of 4-hydroxy-tamoxifen (4-OHT) or fulvestrant in the presence or absence of estrogen (Fig. 6a–c; Supplementary Fig. 8). In the absence of estrogen, both wt-MCF7 and wt-SUM44 showed little sensitivity to fulvestrant, as expected. SUM44-LTED and both MCF7-LTED derivatives were sensitive to fulvestrant in the absence of estrogen confirming ESR1 ligand independence, irrespective of mutation state. In the presence of estrogen, sensitivity to both 4-OHT and fulvestrant was reduced in the low concentration range in SUM44-LTED compared to wt-SUM44. However, while ESR1^Y537S^ was not inhibited by 4-OHT, it was by fulvestrant. Wt-MCF7, MCF7-LTED^Y537C^, and MCF7-LTED^wt^ all showed similar sensitivity to 4-OHT. However, MCF7-LTED^Y537C^ in the presence or absence of estrogen showed greater sensitivity to fulvestrant compared to MCF7-LTED^wt^. The sensitivity of the MCF7-LTED^Y537C^ model to the antiproliferative effect of fulvestrant was further supported in vivo (Fig. 6d).

We subsequently assessed response to drugs inhibiting pathways associated with endocrine resistance such as mTORC (RAD001), ERK1/2 (U0126), and ERBB2/EGFR (lapatinib)^2^. SUM44 derivatives were resistant to the antiproliferative effects of lapatinib and U0126 and showed similar sensitivity to RAD001. The MCF7 derivatives revealed limited response to lapatinib. MCF7-LTED^Y537C^ and wt-MCF7 showed a similar response to RAD001 but not U0126, where MCF7-LTED^Y537C^ showed marked sensitivity in keeping with the increased levels of pERK1/2 in this cell line. The MCF7-LTED^wt^ showed little antiproliferative response to any of the agents tested, suggesting this cell line has a high degree of kinase plasticity (Supplementary Fig. 11a, b).

## Discussion

Acquired resistance to endocrine therapy is a major clinical problem and the elucidations of pathways associated with relapse are of paramount clinical importance to facilitate improvement in treatment. While somatic mutations in *ANDR* have been strongly linked with lack of response to hormone therapy and/or agonist response to anti-androgens in prostate cancer, it is only recently that the importance of *ESR1* mutations in BC has been reported (reviewed by Jeselsohn et al.^7^). In vitro studies using ectopic expression cassettes suggest that the most commonly found mutations, Y537S and D538G, confer ligand independence and exhibit reduced sensitivity to tamoxifen and fulvestrant^11,12^.

We describe for the first time the identification of naturally occurring *ESR1* mutations in ESR1-positive BC cell lines. Importantly, we show that estrogen depletion selects for cells harboring *ESR1* mutations, resulting in estrogen-independent growth and expression of the ESR1 transcriptome. We believe that normal culturing of BC cell lines in the presence of estrogen obviates the need for *ESR1* mutations and that only with the strong selective pressure imparted by culturing in estrogen-depleted medium are alternative growth pathways, including *ESR1* mutations enriched. Furthermore, estrogen deprivation appears to be the primary point for enrichment, as *ESR1* mutated cells did not appear to be augmented during acquisition of resistance to tamoxifen or fulvestrant in vitro. This observation is analogous to our recent clinical study in which *ESR1* mutations in ctDNA of metastatic BC patients were found almost exclusively in patients that had become resistant to AI treatment^10,13^. Additionally, treatment with fulvestrant in vitro appeared to enrich for the pre-existing Y537C mutation (MCF7-LTED-ICIR).

ChIP-seq analysis suggested that ESR1^Y537S^ functions in a ligand-independent manner, largely recapitulating the estrogen-bound-ESR1^wt^ cistrome, which was demonstrated by the fact that ER binding sites and their genomic distribution was overwhelmingly similar in wt-SUM44 and SUM44-LTED cells. The Y537S mutation lies near helix 12 (H12), which governs the ligand-regulated actions of ESR1 via AF-2. Recent studies have suggested that Y537S enables H12 to undergo a conformational change exposing the AF-2 cleft, facilitating recruitment of coregulators in the absence of hormone, leading to further stabilization of H12. In the same study, it was shown that Y537S also increased affinity for AIB1^25^. Assessment of the ESR1^Y537S^ interactome using RIME showed no increase in the association of the naturally occurring mutant ESR1 with AIB1, but did show increased association with FOXA1 and GREB1. One possible explanation for this difference is that the structural studies analyzed only the ESR1 LBD and nuclear receptor interacting domain of AIB1^25^ and thus cellular context was not explored.

Despite this compelling data, indicating the mutant *ESR1* is sufficient to drive adaptation to estrogen deprivation, the cell lines, similar to clinical samples, are heterozygote for both wt and ESR1 mutant alleles. As such, we cannot conclusively differentiate between binding events due to wt and mutant ESR1, so it is possible that the wt allele predominates in LTED. However, there is no evidence in clinical samples that all ESR1 alleles are mutated in metastatic BC cases^11,12,26–29^. Moreover, MCF7^Y537S^ cells, generated by CRISPR-Cas9-mediated knockin mutagenesis, which are heterozygote for ESR1^Y537S^ and express both wt and Y537S mutant ESR1, show estrogen-independent recruitment of ESR1 and coactivators to ESR1 binding regions^24^. These cells demonstrate estrogen-independent expression of ESR1 target genes and grow in an estrogen-independent manner, validating the contribution of the Y537S mutation to estrogen independence when co-expressed with ESR1^wt^.

A second caveat is the role of altered kinase signaling pathways that may arise from extended growth in estrogen-depleted culture conditions to generate LTED and post-translational changes that may impact on the resistance phenotype. Our own studies and those of others have shown that altered kinase signaling can lead to ligand-independent activation of ESR1 (reviewed by Ma et al.^2^). Furthermore, ectopic expression of AKT has been shown to alter the genome-wide binding pattern of ESR1^30^ and that EGF induces a transcriptional program distinct from estrogen^31^. However, genomic profiling of SUM44-LTED cells harboring ESR1^Y537S^ did not provide evidence for altered ESR1 binding patterns compared to wt-SUM44. Second, the CRISPR-Cas9-derived MCF7^Y537S^ cells showed estrogen independence in the absence of prolonged culturing in estrogen-depleted conditions. Finally, CRISPR-Cas9 editing of the Y537C allele re-established estrogen dependence in MCF7-LTED^∆537C^ cells, demonstrating a requirement for the Y537C mutation for the estrogen independence. Taken together, our results support the notion that activating mutations in the *ESR1* are sufficient for driving acquired resistance that does not necessitate changes in other signaling pathways.

Moreover, our in vitro data indicate that ESR1^Y537S/C^ mutations are responsive to fulvestrant, as ESR1 protein expression was downregulated (Fig. 6c), although suppression of growth was less pronounced at low concentrations of the drug, indicating partial resistance of ESR1^Y537S^ but not ESR1^Y537C^. Nonetheless, at the predicted clinically achievable concentrations of fulvestrant^32,33^, ESR1^Y537S^ was as equally sensitive as the ESR1^wt^. This is in keeping with our previous clinical data, which suggests patients harboring an *ESR1* mutation show longer progression-free survival when treated with fulvestrant vs. exemestane^13^. However, in contrast to Y537C, Y537S showed reduced sensitivity to 4-OHT. One explanation for these observations is that, 4-OHT causes Y537S to stabilize H12 by the formation of a hydrogen bond between 537S and E380, effectively reducing the potency of the drug. In contrast, binding of fulvestrant disorders H12. As such, some of the new SERM/SERD agents with enhanced pharmacokinetics capable of increasing the dynamics of H12 may show increased potency against this mutation^25^.

Interestingly, MCF7-LTED^wt^ show evidence of reduced ESR1 activity, with lower expression of estrogen-regulated genes such as *PGR* and increased expression of genes associated with anti-apoptotic activity^34^. Unexpectedly, LTED cells expressing ESR1^wt^ were also less sensitive to fulvestrant compared to ESR1^Y537C^. One explanation is that these cells already have elevated kinase activities and are thus less dependent on ESR1, highlighting once again the complexity of cellular context as well as mutation status on response to endocrine therapy.

Recent genetic studies that have identified *ESR1* mutations in metastatic, endocrine-resistant BC indicate that these mutations result from the selective pressure imposed by inhibition of ESR1 activity by hormonal therapies. The results presented here provide support for this hypothesis. The independent BC cell line models identified here also provide an important resource for studying the relative contribution of *ESR1* mutations and alterations in other signaling pathways, that lead to endocrine resistance. Indeed, the genomic studies described herein provide support for the importance of kinase signaling cascades that have already been implicated in endocrine resistance by our studies, as well as those of other investigators. Our findings demonstrate that *ESR1* mutations provide an important, albeit not the only driver of acquired endocrine resistance, concordant with the clinical observation that ~20% of metastatic tumors harbor mutant *ESR1*. Using resistance models featuring *ESR1* mutations and those that do not involve *ESR1* mutations should prove to be valuable in aiding patient management, and for assessing new treatment approaches for endocrine-resistant BC. We and others will need to consider the presence and any phenotypic effects of these and possibly other acquired/selected mutations when using these derived cell lines for mechanistic or pharmacological studies and interpreting data from them.

## Methods

### Reagents

Following antibodies were used for immunoblotting: pESR1^ser167^ (CST cat-5587, 1:1000), pESR1^ser118^ (CST cat-2511, 1:1000), total-ESR1 (Santa Cruz sc8002, 1:800 or Novacastra (NCL-ER-6F11), 1:1000), total-FOXA1 (Abcam Ab23738, 1:1000) total-PGR (Novocastra NCL-L-PGR, 1:500 or Santa Cruz sc-538, 1:200), pERBB2 (CST-2243, 1:1000), total-ERBB2 (CST-4290, 1:1000), pEGFR (CST-3777, 1:1000), total-EGFR (CST-2232, 1:1000), pAKT^ser437^ (CST-9271, 1:1000), total-AKT (CST-9272, 1:1000), pERK1/2 (Sigma-Aldrich, 1:2000), total-ERK1/2 (CST-9102, 1:1000), TFF1 (Santa Cruz sc28925, 1:200), RARA (Abcam Ab39971, 1:1000), cathepsin D (CTSD) (Abcam Ab6313, 1:2000), actin (Abcam Ab6276, 1:10,000), and tubulin (Sigma T-9026, 1:2000). Secondary antibodies (horseradish peroxidase-linked, 1:2000) were obtained from Dako. For ChIP, the following antibodies were used: ESR1 (Santa Cruz sc543), CBP (Santa Cruz sc369), and FOXA1 (Abcam Ab23738). 17-β-estradiol (E) and 4-hydroxytamoxifen (4-OHT) were purchased from Sigma-Aldrich and fulvestrant (ICI182780) from Tocris Bioscience.

### Cell culture

Wt-MCF7, wt-HCC1428, wt-ZR75.1, and wt-SUM44 were purchased from the ATCC and Asterand. Cell lines were banked in multiple aliquots upon receipt to reduce risk of phenotypic drift and identity confirmed by short tandem repeats (STR) profiling. All cell lines were routinely screened for mycoplasma contamination. Wt cell lines were cultured in phenol red-free RPMI supplemented with 10% fetal bovine serum (FBS) and exogenous estradiol (1 nM). The respective LTED derivatives were cultured, as previously described^14,15^ in phenol red-free RPMI supplemented with 10% dextran charcoal stripped FBS (DCC medium). ICI-R and TAMR cell lines were cultured in their respective basal medium supplemented with 100 nM fulvestrant (ICI182780) or 100 nM 4-OHT. All experiments were performed under basal conditions unless otherwise stated.

### Proliferation assays

Proliferation assays were performed as previously described for experiments involving drugs and siRNA studies^14,15^. In summary, cells were deprived of estrogen for 48–72 h prior to treatment with On-target plus siRNA for human-si*FOXA1* or non-targeting pool (sicontrol) (Thermo scientific, Dharmacon). Knockdown efficacy was determined by qRT-PCR. For drug studies, cells were treated for 6 days with a medium change at day 3, as previously described^14^. To analyze growth over time, cells were cultured as detailed above in DCC medium with or without estradiol and data recorded using an IncuCyte ZOOM live cell analyzer (Essen Biomedics). Three images per well were taken every 12 h over a 6-day period.

### qRT-PCR

RNA was extracted using the RNeasy kit (Qiagen), quantified and reverse-transcribed with SuperScriptIII First Strand Synthesis System (Invitrogen). Taqman gene expression assays (Applied Biosystems) were used to quantify *TFF1* (Hs00907239_m1 and Hs00170216_m1), *PGR* (Hs00172183_m1), *GREB1* (Hs00536409_m1), *CTSD* (Hs00157201_m1), *ESR1* (Hs00174860_m1), *CCND1* (Hs00765553_m1), and the house-keeping genes *FKBP15* (Hs00391480_m1) and *GAPDH* (Hs99999905_m1). The relative quantity was determined using ΔΔCt, according to the manufacturer’s instructions (Applied Biosystems).

### Exome sequencing

Exome libraries were generated with SureSelect Human All Exon V5 kit and sequenced (paired-end 100 bp) on an Illumina HiSeq2500. Reads were aligned to GRCh37-lite-build37 using BWA mem (v0.7.12-r1039)^35^, sorted with samtools (v1.2)^36^ and further processed using picard tools (http://picard.sourceforge.net) (v1.128) with default parameters. Single nucleotide variants (SNVs) were detected using VarScan v2.3.5^37^ with default parameters (except --mpileup 1, --output-vcf) and wt cell samples as baseline. Multi-mapped reads were excluded and base alignment quality (BAQ) was turned off for pileup with samtools. To get high confidence somatic mutations, SNVs were filtered by using: (i) processSomatic of VarScan with empirically-derived criteria: minimum VAF in LTED cells: 0.10, maximum VAF in wt: 0.05, *p*-value = 0.07; (ii) fpfilter.pl from VarScan together with bam-read count (--min-base-quality 15, --min-mapping-quality 1) to reduce number of false positives. Variants were annotated using SnpEff v4.1 B (http://snpeff.sourceforge.net/SnpEff_manual.html). Mutations were annotated with Tier levels^38^ using BedTools v2.22.1^39^. ascatNGS (https://github.com/cancerit/ascatNgs) was used to generate LogR and BAF values. Data have been deposited in the sequence read archive: BioProject ID PRJNA390496.

### Ion torrent

DNA was amplified using Ion AmpliSeq Library Kit 2.0 (Life Technologies), digested, Ion Xpress.

Barcode adapters ligated and purified with Agencourt AMPure XP magnetic beads (Beckman Coulter). Libraries were quantified by qPCR using an Ion Library Quantification Kit (Life Technologies), templated on the Ion OneTouch2 System (Life Technologies) and sequenced on the Ion PGM System (Life Technologies). Reads were aligned by the PGM server with standard settings to the reference genome hg19, samtools v1.2 was used to calculate the on-target coverage.

IonReporter (v4.4) was used for mutation calling (parameters: data quality stringency = 12, downsample to coverage = 4000, SNP/InDel/MNP min cov each strand = 50, SNP/InDel/MNP min variant score = 15, SNP/InDel/MNP min coverage = 250, hotspot min variant score = 6, hotspot min coverage = 150). All mutations called were manually reviewed in Integrative Genomics Viewer (IGV) and included in the analysis if they had a VAF ≥ 1%.

### ddPCR

ddPCR assays for the *ESR1* mutations Y537S and Y537C using Taqman probes were used as previously described^10^. Very-low-frequency mutations were only considered to be present if two or more FAM-positive droplets were detected in the total of the wt sample.

### Cycle sequencing for validation

*ESR1* mutations were validated by cycle sequencing by eurofins genomics (Eurofins). DNA was amplified using forward primer 5ʹ- AAGTGGCTGCAGGGAGAGT-3ʹ and reverse primer 5ʹ-TGGTGCATGATGAGGGTAAA-3ʹ.

### Fluorescence in situ hybridization

FISH probes hybridizing at 6q25 (ESR1) and chromosome-6 (CEN6) were purchased from Empire Genomics. Cell pellets were fixed in 4% paraformaldehyde and paraffin-embedded. Five-micron sections were subjected to the SwiftFISH rapid hybridization protocol (Empire Genomics), according to the manufacturer’s instructions. Sections were mounted in DAPI-containing Vectashield (Vector). FISH probes signals were analyzed using fluorescent microscope (Leica).

### RNA-seq

Libraries were created after Ribo-zero rRNA Removal Kit (Illumina) using NEBNext Ultra Directional RNA (NEB) or Truseq Stranded Total RNA (Illumina) Library Prep Kit and sequenced using the HiSeq2500 (paired-end 100 bp v4 chemistry). Tophat (v2.1) and Cuffdiff (v2.2.1)^40^ using default parameters (GSE100075). K-means clustering was performed using the k-means function in the stats package in R. The number of clusters used was determined by the number of clusters generated in unsupervised clustering using hclust (method = complete) function in R with of a matrix of correlation-based distances using the Spearman method.

### ChIP-seq

ChIP-qPCR and ChIP-seq were performed, as previously described^14,41^. Paired-end 50 bp ChIP-seq data were generated by rapid-mode HiSeq. Reads were aligned to the Human Reference Genome (assembly hg19) using BWA^35^ removing all reads with a quality score < 15. Peaks were called using MACS2 (v2.1.0.20150420)^42^ with default parameters. Only binding events that occurred in two biological replicates were considered differential binding sites using Diffbind v1.14.5^43^ and R v3.2.1. Motif analysis was performed using centrimo (500 bp centered on summit of peak) (http://meme-suite.org/) (GSE100074). Bar charts were generated with ChIPseeker package in R^44^.

### GSEA

Integration of RNA-seq and ChIP-seq diffBind data were carried out using GSEA, as previously described^45^. In summary, all genes assessed using RNA-seq were ranked and weighted by their mean Log2 fold change. Lists of genes that overlapped with regions showing significant differential binding were identified. These data were then analyzed using the GSEA v2.0.13 GSEA Pre-ranked tool. The default setting was applied. Finally, additional analysis of gene sets (e.g., overlaps between significant binding events and closest genes that are significantly differentially expressed) were performed using the Molecular Signature Database (http://software.broadinstitute.org/gsea/msigdb/annotate.jsp) to compute overlaps with Hallmark gene sets that represent well-defined biological states or processes. Significance of overlap between gene sets was determined by hypergeometric test.

### RIME and dimethyl labeling

RIME^22^ and stable isotope dimethyl labeling^21^ were performed, as previously described. The wt-SUM44 and SUM44-LTED were labeled with the medium and light isotope reagent, respectively. Labeled samples were pooled at an approximate 1:1 ratio, dried down and fractionated using 12 cm IPG strip pH 3–10, as previously described^46^. RIME and dimethyl label fractions were desalted (SUM SS18V, The Nest Group Inc) and run through LC-MS/MS using LTQ Velos Orbitrap MS. The data acquisition mode was set, as previously described^46^. Raw data for RIME and dimethyl labeling were analyzed using MaxQuant 1.5.1.0^46,47^. Search parameters were as previously described^46^. All proteomics data are deposited within the PRIDE database (PXD004807).

### Identification of mutation at protein level using ddMS2/PRM

ESR1-RIME samples were subjected to ddMS2-PRM analysis in order to verify the presence of wt and mutated serine or cysteine in the SUM44-LTED and MCF7-LTED samples, respectively (Supplementary Data 3). The analysis was performed using a Q-Exactive HF mass spectrometer (Thermo Scientific, Hemel Hempstead, UK). For each analysis, three biological replicates with two technical replicates were run. Heavy peptides were purchased from Thermo Fischer Scientific (PEPOTEC, grade 3). Reversed phase chromatography was performed on a Dionex UltiMate 3000 RSLC nano system (Thermo Fisher Scientific, Hemel Hempstead, UK) using an Acclaim PepMap100 C18 trap cartridge (0.5 mm i.d. × 5 mm, 5 µm bead size, 100 Å pore size; loaded in a bi-directional manner). Peptides were resolved on a 75 µm I.D. 50 cm C18 Easy-Spray packed emitter column (2 µm particle size; PepMap RSLC, Thermo Scientific, Hemel Hempstead, UK) over 90 min using a three-step gradient of 96:4 to 50:50 buffer A:B (*t* = 0 min 4% B, 0.5 min 4% B, 12.0 min 10% B, 43.0 min 25% B, 90.0 min 50% B) (buffer A: 2% acetonitrile/0.1% formic acid; buffer B: 80% acetonitrile/0.1% formic acid) at 250 nl per min. Peptides were iodized by electrospray ionization using 1.8 kV applied using the Easy-Spray ion Source. Sample was infused into the mass spectrometer directly from the packed emitter (5 µm exit bore). The ion transfer tube was heated to 275 °C and the S-lens set to 50%. MS/MS were acquired using parallel reaction monitoring (PRM) and data-dependent (ddMS2) acquisitions based on a full FT-MS scan from 350 to 1850 *m/z* at 120,000 resolution, with a target automatic gain control (AGC) value of 3,000,000 and a maximum injection time of 50 ms. No internal lock mass calibrant was used. Eight PRM scans were triggered (FT-Orbitrap scans at 30,000 resolution, AGC target 2e5, 100 ms maximum injection time, normalized collision energy 35) if an ion from scheduled inclusion list was present. Then, the top five most intense ions were fragmented by higher energy collision-induced dissociation and dynamically excluded for 20 s (FT-Orbitrap scans at 30,000 resolution, AGC target 1e5, activation time 10 ms, 50 ms maximum injection time, normalized collision energy 28, selected first mass at 140 *m/z*). Precursor ions with unknown or single charge states were excluded from selection. Data analysis of raw MS/MS was carried out using Mascot V2.3 via Proteome Discoverer v1.4. Peak lists were searched against the human Uniprot FASTA database (20,305 sequences) containing the wt and mutant sequence. Spectra were searched for a match to fully-tryptic peptides with up to two missed cleavage sites. Search parameters were chosen as follows: serine/threonine phosphorylation, protein N-terminal acetylation, peptide N-terminal glutamine to pyroGlu, and oxidation of methionines were all considered as variable modifications, whereas cysteine carbamidomethylation was selected as a fixed modification. Precursor ion mass tolerance was set to 15 ppm for the first search, fragment ion mass tolerance for ion analyzed spectra was set to 0.02 Da. Resulting peptide and protein lists were grouped and validated using Scaffold v4 (Proteome Software Inc., Portland, OR). Protein identifications were automatically accepted if they contained at least two unique peptides assigned at 1% FDR. The raw data have been deposited in Passel (PASS01062).

### Immunoblotting

Whole-cell extracts were generated from cells cultured under basal conditions or DCC medium with or without the addition of estrogen for comparative studies where noted. Equal amounts of protein were resolved by SDS–PAGE and subjected to immunoblot analysis. Antigen–antibody interactions were detected with ECL reagent (Amersham, UK) using the antibodies referred above.

### CRISPR-Cas9-mediated generation of the MCF7-LTED^∆537C^ cells

Gene knockins for a modified *ESR1* exon 8, encoding a wt open reading frame with silent mutations to facilitate PCR analysis, were made using CRISPR-Cas9-mediated homologous recombination in MCF7-LTED^Y537C^ cells. ESR1 gene targeting was carried out using CRISPR 4834093 (5ʹ-GAGTGCTGAAATCCCTAGAA-3ʹ) cloned into a guide-RNA expression plasmid (a gift from George Church; Addgene plasmid #41824), as described previously^24^. The target sequence for this CRISPR is located in intron 7, on the antisense strand, 73 nt from the start of *ESR1* Exon 8. For making the gene knockin, a previously described *ESR1* exon 8 Y537S gene targeting donor construct^24^ was modified by site-directed mutagenesis to change codon 537 from Serine (TCT) to Tyrosine (TAT), as found in the wt sequence. Additional mutations, to destroy the PAM for CRISPR 4834093, were made by changing a run of four C nucleotides, located 77 nt 5ʹ to the start of *ESR1* Exon 8, to four G nucleotides. Genome editing, detection of gene targeting events, and sequence characterization of gene targeted alleles were carried out as described previously^24^, with the exception that following transfection, cells were recovered in full medium supplemented with 10% FCS, and Exon 8 knockin clones identified through stochastic cloning.

### Energy phenotype and Mito Fuel Flex analysis

SUM44 and MCF7 cells were plated at a confluency of 1.0 × 10^4^ per well in a 96-well Seahorse cell culture microplates and incubated in a 5% CO_2_ incubator at 37 °C overnight. The next morning, culture media was replaced with pH-adjusted (pH = 7.4 ± 0.1) bicarbonate-free DMEM with 10 mM glucose, 1 mM sodium pyruvate, and 2 mM l-glutamine. The plate was then incubated at 37 °C for 1 h in a non-CO_2_ incubator. For the Mito Fuel Flex test, oxygen consumption rates were measured using the Seahorse XF Mito Fuel Flex Test Kit (Agilent, 103260-100) on an XFe96 Analyzer. Cell numbers were normalized using CyQuant (Thermo Fisher, C35012).

### Cell migration assay

Cells growing in basal media were washed several times with phenol red-free RPMI1640 containing 1% DCC-FBS. A total of 2.5 × 10^4^ cells were seeded into the upper chambers of Corning FluoroBlok 96-multiwell insert system plates (Corning, UK). The lower chambers were filled with RPMI1640 containing 1% DCC-FBS plus 100 ng/ml human recombinant EGF, as chemo-attractant, and plates were incubated at 37 °C. After 16 h, the medium was removed from the lower chambers and wells were washed with PBS. PBS containing 1 μM calcein AM (Invitrogen) was added to the lower chambers and the plates were incubated at 37 °C for 30 min. Fluorescence intensity was measured from the bottom of the plates using a 490 nm excitation filter and a 520 nm emission filter in a Victor X5 plate reader (PerkinElmer). Data are expressed as the mean of eight technical replicates.

### Human tumor xenografts modeling relapse on AI therapy

In vivo studies were carried out in ovariectomized 8- to 12-week-old female BALB/c nude mice in accordance with Home Office Guidelines and approved by the Institute of Cancer Research Ethics Committee. Xenografts modeling patients resistant to AI were initiated by innoculating MCF7-LTED^Y537C^ (10^7^) cells in basement membrane matrix (Matrigel; BD Biosciences) into the right flank of each animal. Once tumors reached 7 mm in size, they were size matched and mice treated with either 5 mg per kg fulvestrant once per week or vehicle control. The study operator was blinded to treatment. Tumor growth was assessed twice weekly in both arms by caliper measurements of the two largest diameters. Volumes were then calculated according to the formula: *a* × *b* ^2^  × π/6, where *a* and *b* are orthogonal tumor diameters. Tumor volumes were then expressed as median relative fold change in volume at the start of treatment. At the end of study, data were available for seven animals in the control arm and nine animals in the fulvestrant treatment arm. Overall statistical differences between the treatment and control arms were calculated using an unpaired *t*-test.

### Statistics analysis

Statistical methodologies pertinent to each method are held within the sections above.

### Data availability

The data supporting the finding from this manuscript have been deposited as follows. Whole-exome sequencing has been deposited in the sequence read archive BioProject ID PRJNA390496. RNA-seq and ChIP-seq data have been deposited with the NCBI gene expression omnibus (GEO) (http://ncbi.nlm.nih.gov/geo/): ChIP-seq data for wt-MCF7, MCF7-LTED^wt^, MCF7-LTED^Y537C^ wt-SUM44 and SUM44-LTED (GSE100074), RNA-seq (GSE100075) for wt-MCF7, MCF7-LTED^wt^, MCF7-LTED^Y537C^, wt-SUM44 and SUM44-LTED, CRISPR-cas9 MCF7^Y537S^ ChIP-seq and RNA-seq data (GSE78286)^24^. All proteomics data sets are deposited within the PRIDE database (PXD004807) or Passel (PASS01062) for targeted sequencing.

## Electronic supplementary material

Supplementary Information

Description of Additional Supplementary Files

Supplementary Data 1

Supplementary Data 2

Supplementary Data 3
