# Supplementary material for: Discovery of naturally occurring ESR1 mutations in breast cancer cell lines modelling endocrine resistance
Source: Nat Commun. 2017 Nov 30;8:1865. doi: 10.1038/s41467-017-01864-y (PMC5709387; doi:10.1038/s41467-017-01864-y)
Supplement: Supplementary file 1 — Supplementary Information [file 41467_2017_1864_MOESM1_ESM.pdf]

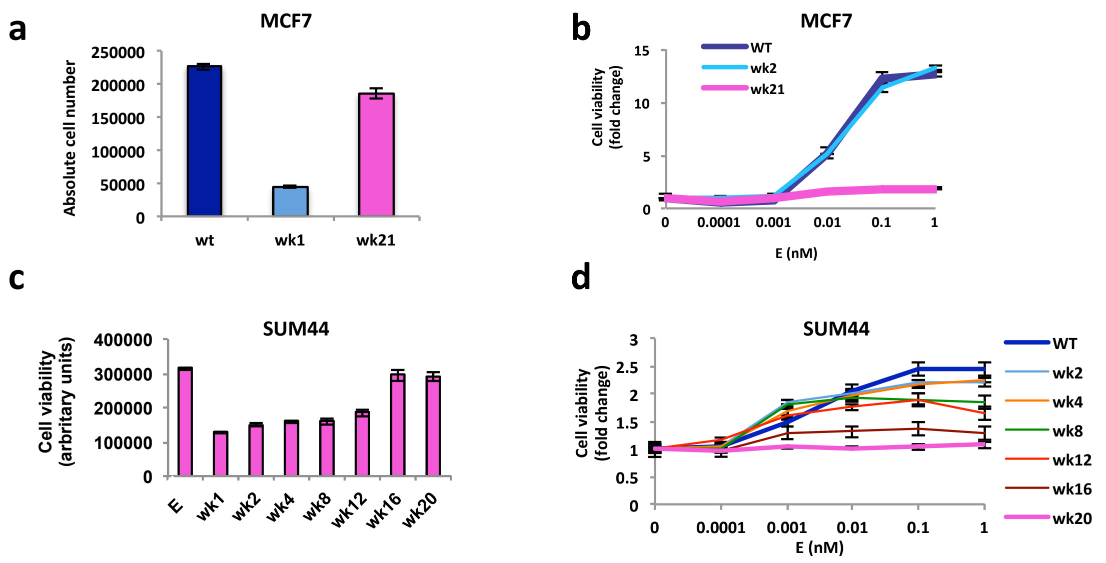

Supplementary Figure 1 (a) Proliferation rate in the absence of exogenous E was assessed at week 1 and 21 and compared to wt-MCF7 cultured under basal condition in the presence of E (estradiol) (1nM). Cell numbers were recorded at day zero and after 6 days growth. Data is expressed as absolute cell number. (Data is representative of n=3 biological experiments. Each assay consisted of n=8 technical replicates). Bars represent mean ± SEM. **(b)** Comparison of the proliferation of MCF7 cells to increasing concentrations of E showing ligand-independent growth after 21 weeks of estrogen-deprivation. Cells were treated for 6 days as indicated. Cell viability was assessed using cell TitreGlo. Bars represent mean ± SEM. **(c)** Characterisation of SUM44-LTED showing acquisition of a ligand-independent phenotype over time. Cells were treated for 6 days. Cell viability was assessed using cell TitreGlo. Bars represent mean ± SEM. **(d)** During acquisition of resistance to long-term estrogen deprivation SUM44 cells show a reduction in response to exogenous E. (n=8 technical replicates at each given time point during acquisition of estrogen-independence)

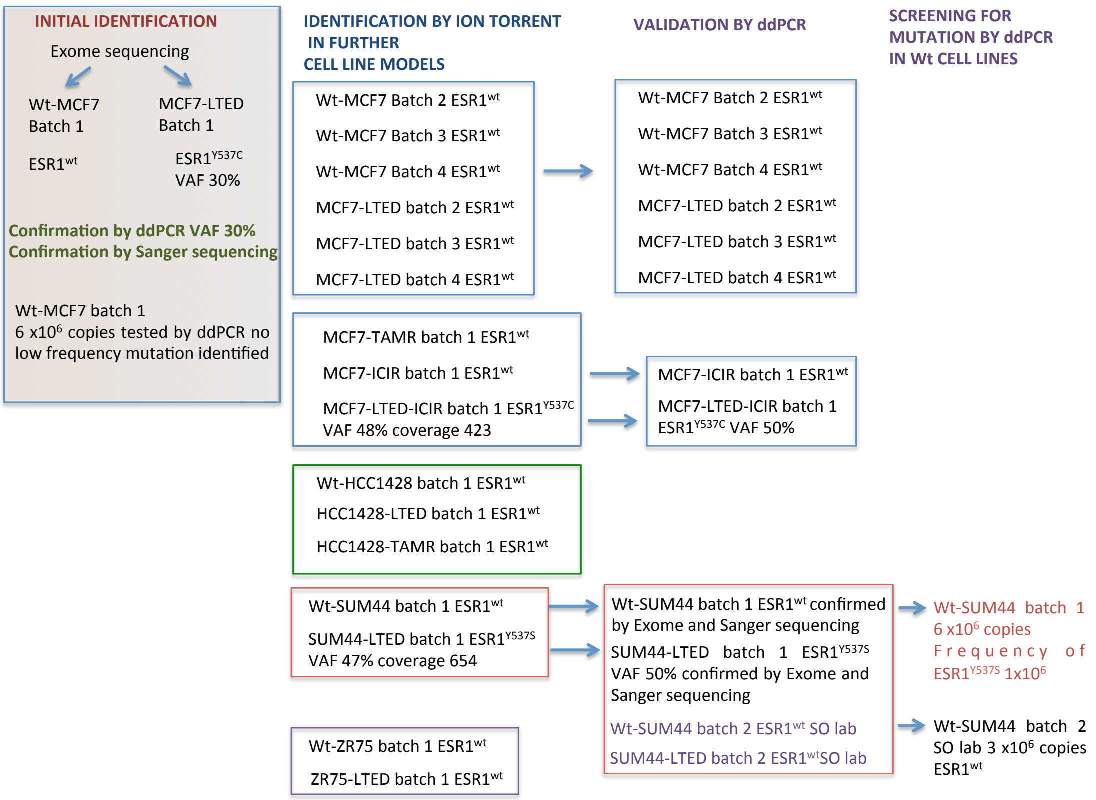

Supplementary Figure 2 Flow diagram showing the cell lines and history of screening procedures to identify natural occurring ESR1 mutation. Data shows variant allele frequency (VAF) and coverage.

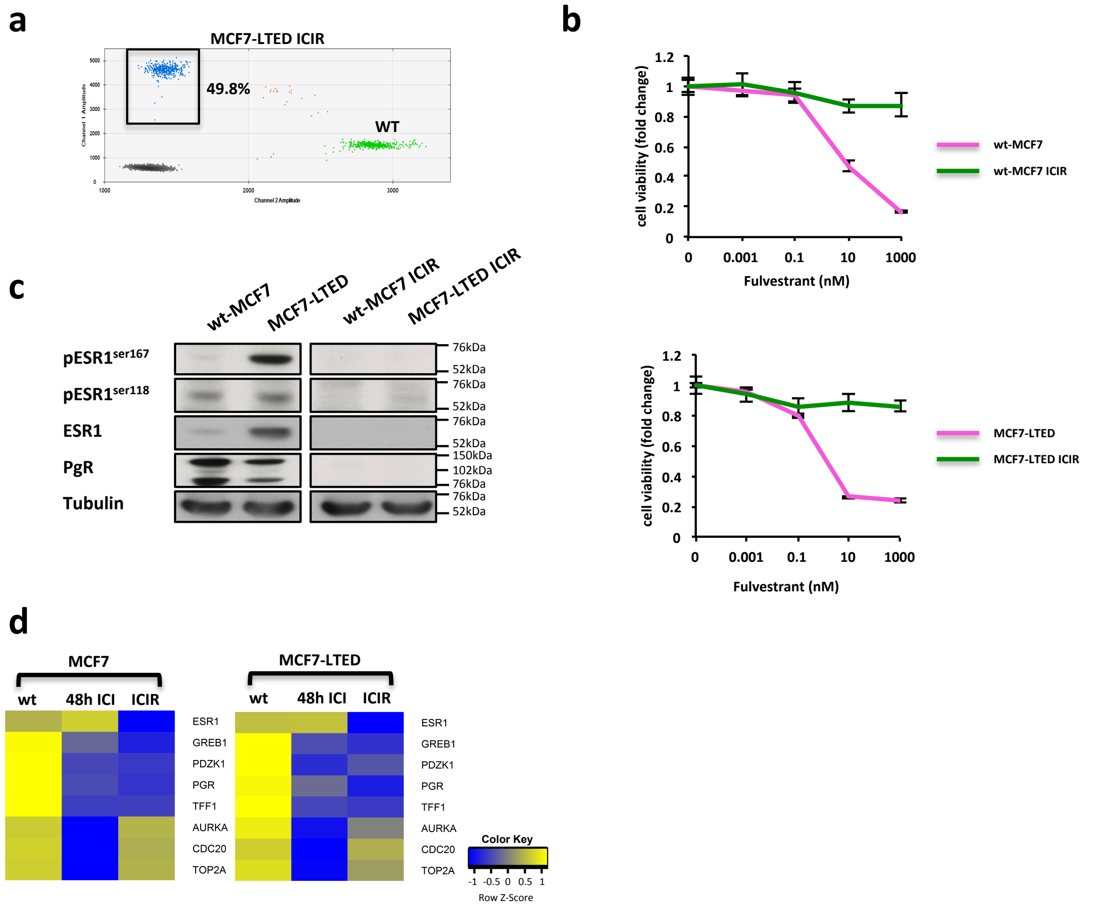

Supplementary Figure 3 (a) ddPCR validation of *Y537C* mutation in MCF7-LTED-ICIR cells. **(b)** Proliferation assays showing response of MCF7, MCF7-ICIR, MCF7-LTED and MCF7-LTED-ICIR to escalating concentrations of fulvestrant (ICI). Cells were treated for 6 days. Cell viability was assessed using cell TitreGlo. Bars represent mean ± SEM. Data is representative of n=4 biological replicates with 8 technical replicates. **(c)** Comparative western blot showing alterations in expression of key proteins in response to acquisition of resistance to LTED and fulvestrant (ICI). MCF7-LTED show increased levels of ESR1, which is phosphorylated at ser^{118} and ser^{167}. Both MCF7-ICIR and MCF7-LTED-ICIR show loss of *ESR1* and progesterone receptor (*PGR*) expression. **(d)** Comparison of the expression of endogenous estrogen-regulated genes. Wt-MCF7 and MCF7-LTED were treated ± fulvestrant (ICI) for 48 hours and alteration in gene expression compared with their respective ICIR derivatives.

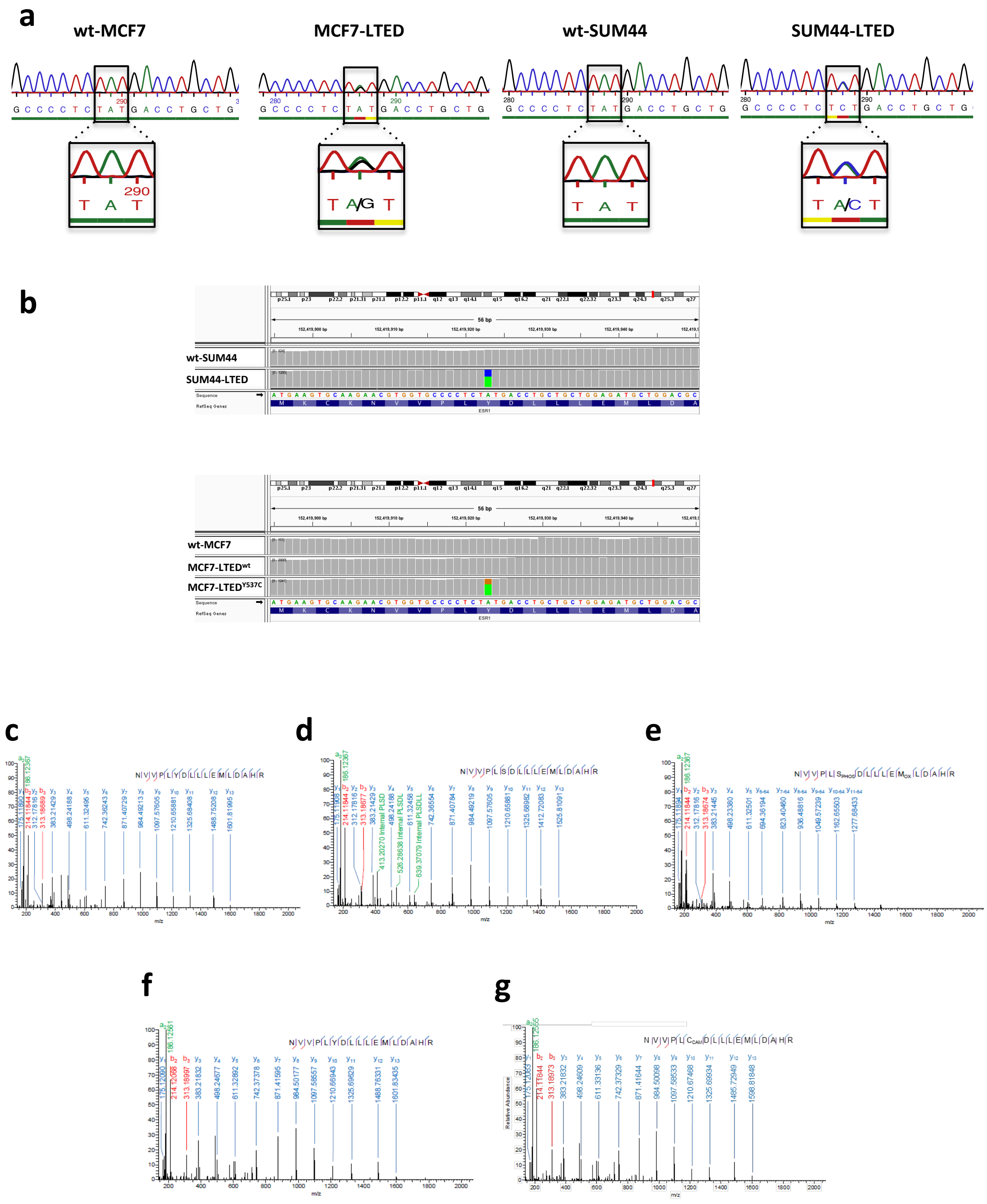

Supplementary Figure 4 (a) Sanger sequencing of wt-MCF7, wt-SUM44 and their associated LTED derivatives confirming the mutations within the adapted cell lines. **(b)** RNA-seq analysis confirms that the ESR1 mutations are transcribed. Identification of the wt (tyrosine) and the mutated peptides (serine or cysteine) using mass spectrometry**:** (**c**) MS/MS spectrum (raw file QE02689, spectrum 30334, scans 39034) of the wt peptide (K)532-NVVPLYDLLLEMLDAHR-548(L) (precursor ion 671.0263^{3+}, error -1.973 ppm, ion score 90.17) **(d)** MS/MS spectrum (raw file QE02689, spectrum 29189, scans 37856) of the mutated peptide (K)532-NVVPLSDLLLEMLDAHR-548(L) (precursor ion 645.6837^{3+}, error -0.2263 ppm,

ion score 74.23). (**e**) MS/MS spectrum (raw file QE02688, spectrum 27513, scans 36476) indicating phosphorylation at S537 in the phosphopeptide (K)532-NVVPL**S**DLLLEM$_{(ox)}$LDAHR-548(L) (precursor ion: 677.6709^{3+}; error -0.07111 ppm, mascot ion score 41.35). (**f**) MS/MS spectrum (raw file QE02832, spectrum 31427, scans 40455) of the wt peptide (K)532-NVVPLYDLLLEMLDAHR-548(L) (precursor ion 671.0339^{3+}, error 9.304 ppm, ion score 73.21) (**g**) MS/MS spectrum (raw file QE02832, spectrum 30563, scans 39551) identifying the carbamidomethylated C537 mutant in the peptide (K)532-NVVPL**C**DLLLEMLDAHR-548(L) (precursor ion: 670.0222^{3+}, error 8.166 ppm, mascot ion score 72.87). Major peaks only assigned for clarity. Data is representative of n=3 biological experiments with 2 technical replicates within each.

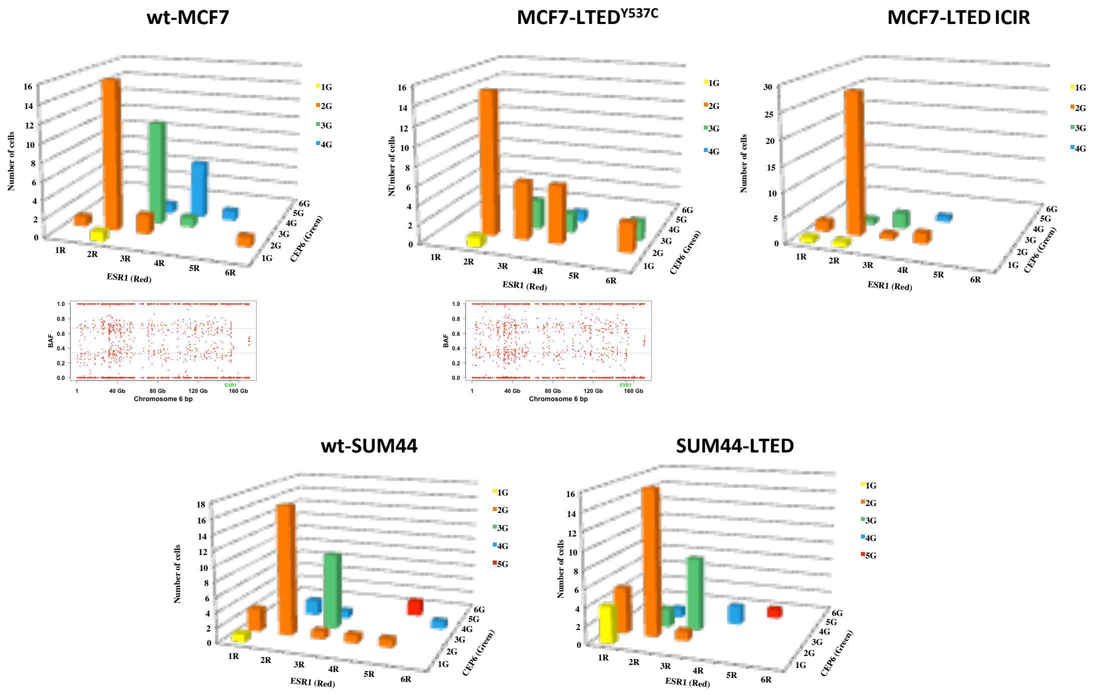

Supplementary Figure 5 Histogram showing alterations in ESR1 and centromere (CEN6) copy number in MCF7 and SUM44 endocrine resistant derivatives together with allelic imbalance represented by B-allelle frequency (BAF) from exome sequencing shown for wt-MCF7 and MCF7-LTED^{Y537C}.

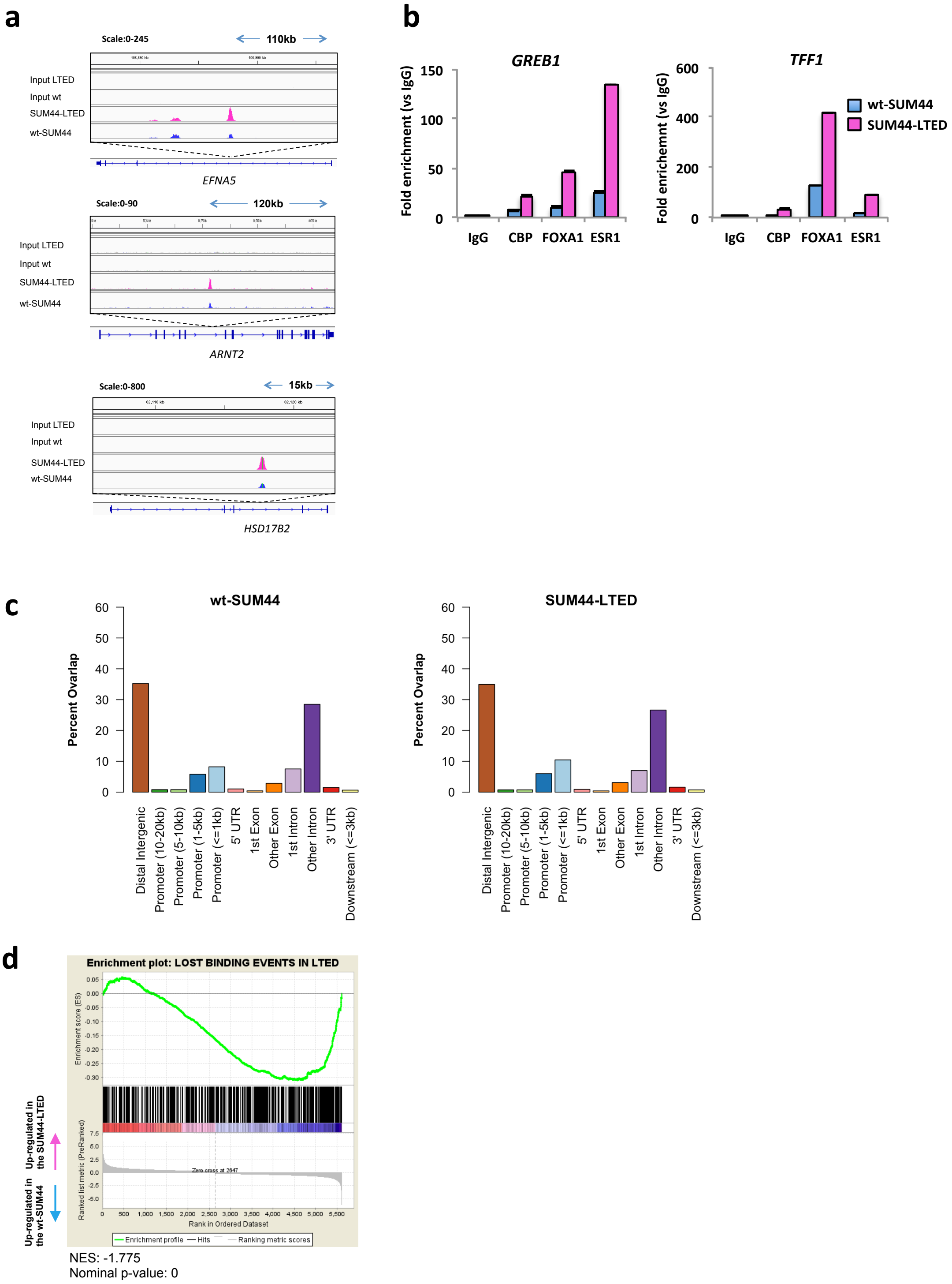

Supplementary Figure 6 Characterisation of SUM44-LTED. **(a)** IGV visual validation of selected peaks differentially bound by *ESR1*^{Y537S}. **(b)** ChIP validation of CBP, FOXA1 and ESR1 binding to the *GREB1* and *TFF1* promoters in wt-SUM44 and SUM44-LTED (n=2 biological replicates). **(c)** Bar graph highlighting genome distribution of ESR1 binding sites in wt-SUM44 and SUM44-LTED. **(d)** GSEA was conducted comparing RNA-seq with loss of ESR1^{Y537S} binding events in SUM44-LTED.

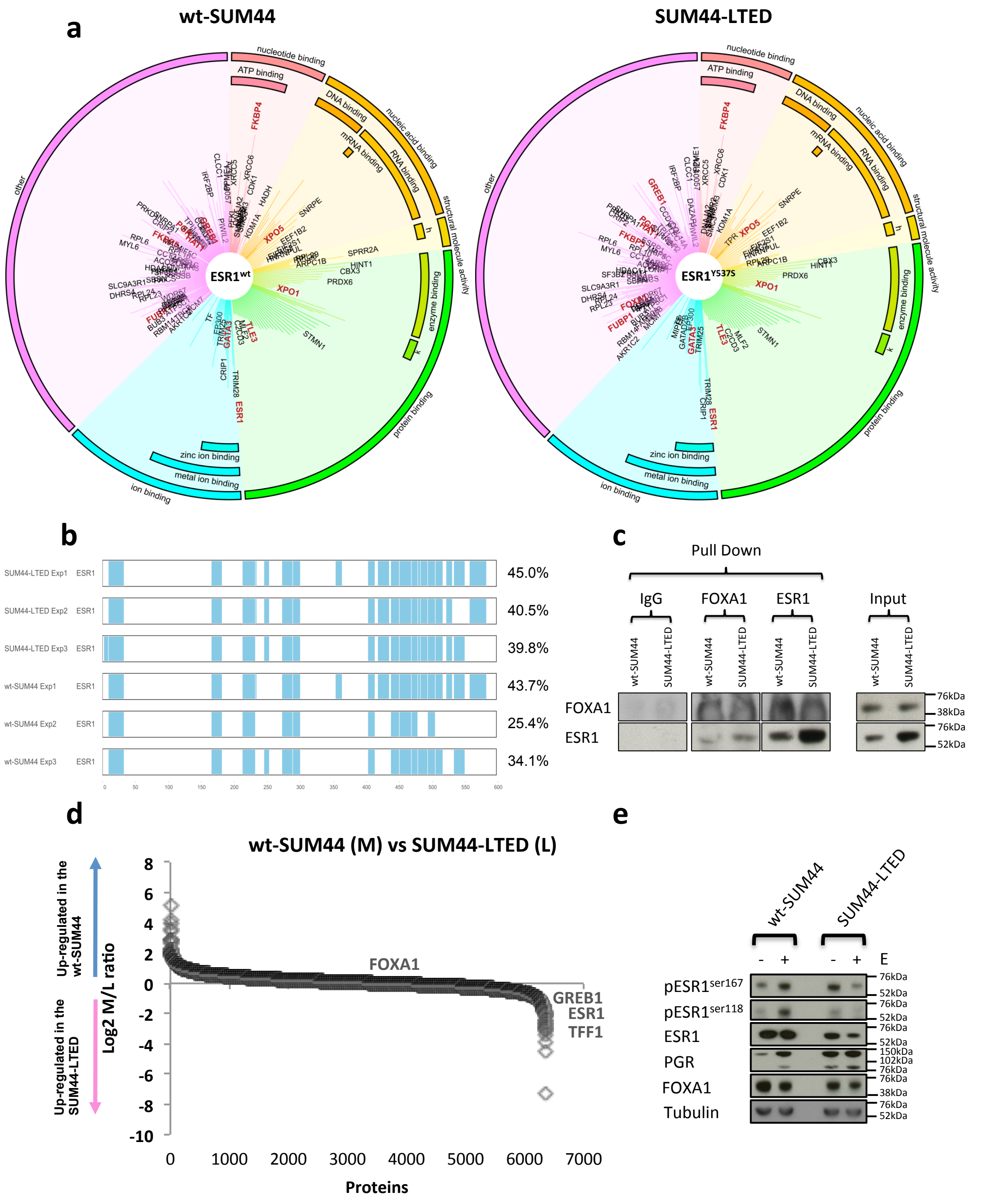

Supplementary Figure 7 (a) MS-ARC plots depicting ESR1 RIME data conducted in wt-SUM44 and SUM44-LTED showing ESR1-associated proteins, which were filtered for non-specific interactions using IgG controls (n=2 biological replicates). The clustering is ordered according to molecular function. The length of the line represents ranking of identified proteins based on sequence coverage. Proteins shown in red are known to interact with ESR1. **(b)** ESR1 sequence coverage from three biological RIME experiments. **(c)** Comparative immunoprecipitation of ESR1, FOXA1 and IgG in wt-SUM44 and SUM44-LTED cells. **(d)** Comparative dimethyl-

labelling of wt-SUM44 versus SUM44-LTED showing increased abundance of ESR1, TFF1 and GREB1 but not FOXA1 in SUM44-LTED. The data shown is from 2 biological replicates and 11 fractions. (**e**) Immunoblot showing the alterations in ESR1 abundance and phosphorylation of ESR1 in wt-SUM44 and SUM44-LTED cultured in DCC medium with or without E.

Figure 3b

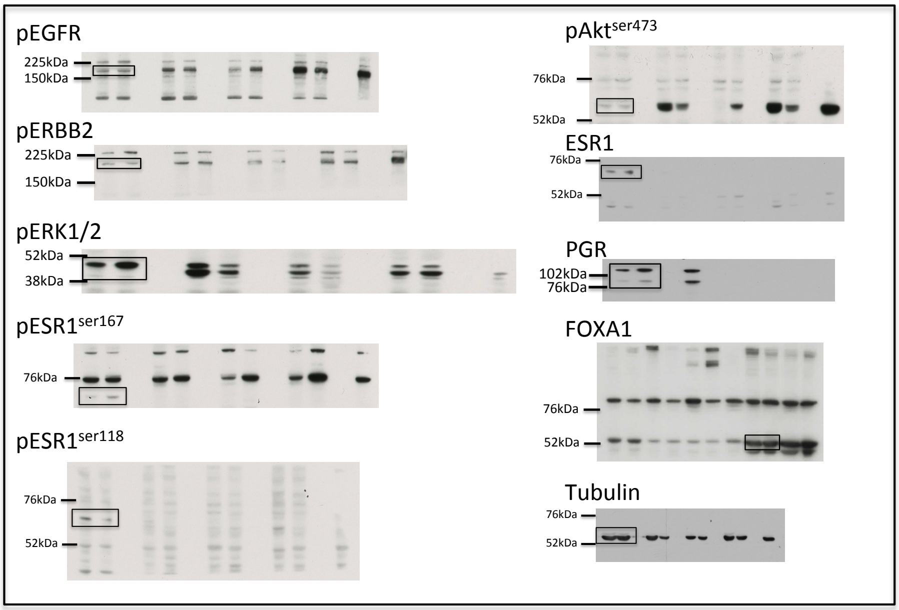

Figure 4b

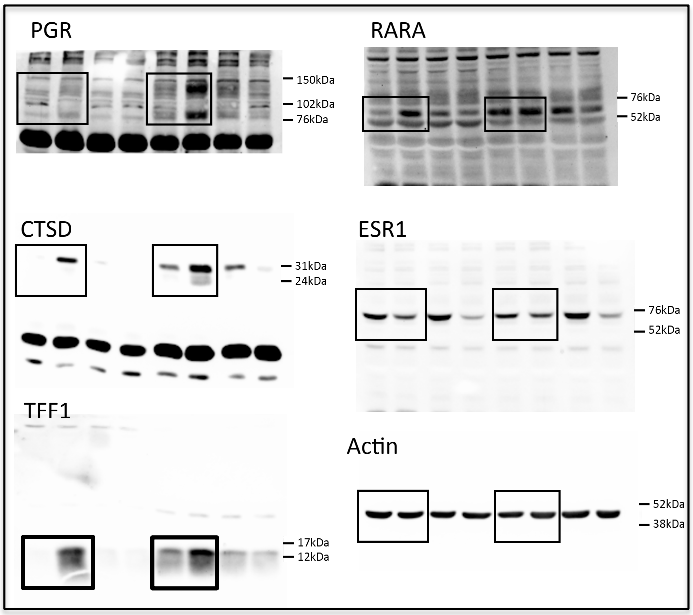

Figure 6c

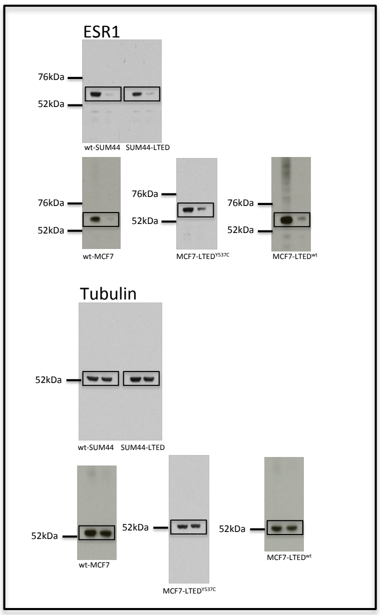

Supplementary Figure 8. Original immunoblots used for figures indicated within the main manuscript

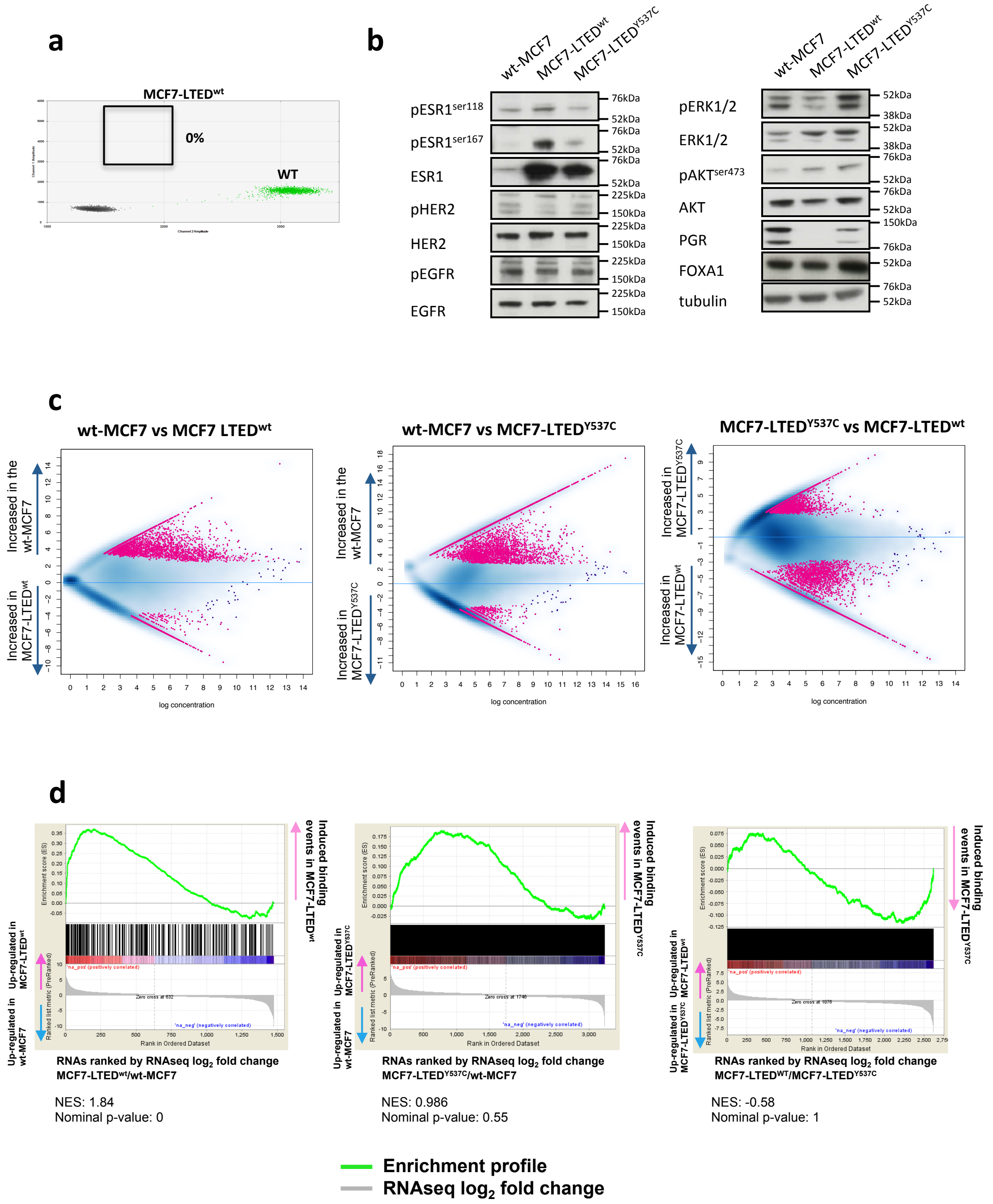

Supplementary Figure 9 (a) ddPCR validation of MCF7-LTED^{wt} showing lack of the Y537C mutation confirming this LTED cell line uses an altered mechanism of resistance. Data shown is representative of n=3 biological replicates.**(b)** Immunoblotting highlighting changes in the abundance of proteins previously associated with endocrine resistant phenotypes. **(c)** MA plots showing the differential binding affinity of ESR1. The x-axis shows log concentration of sequenced tags per peak; y-axis represents log fold change of wt-MCF7/MCF7-LTED^{wt}, wt-MCF7/MCF7-LTED^{Y537C} and MCF7-LTED^{wt}/MCF7-LTED^{Y537C}. **(d)** GSEA was

conducted comparing transcripts with ESR1 induced binding events in wt-MCF7, MCF7-LTED^{wt} and MCF7-LTED^{Y537C}.

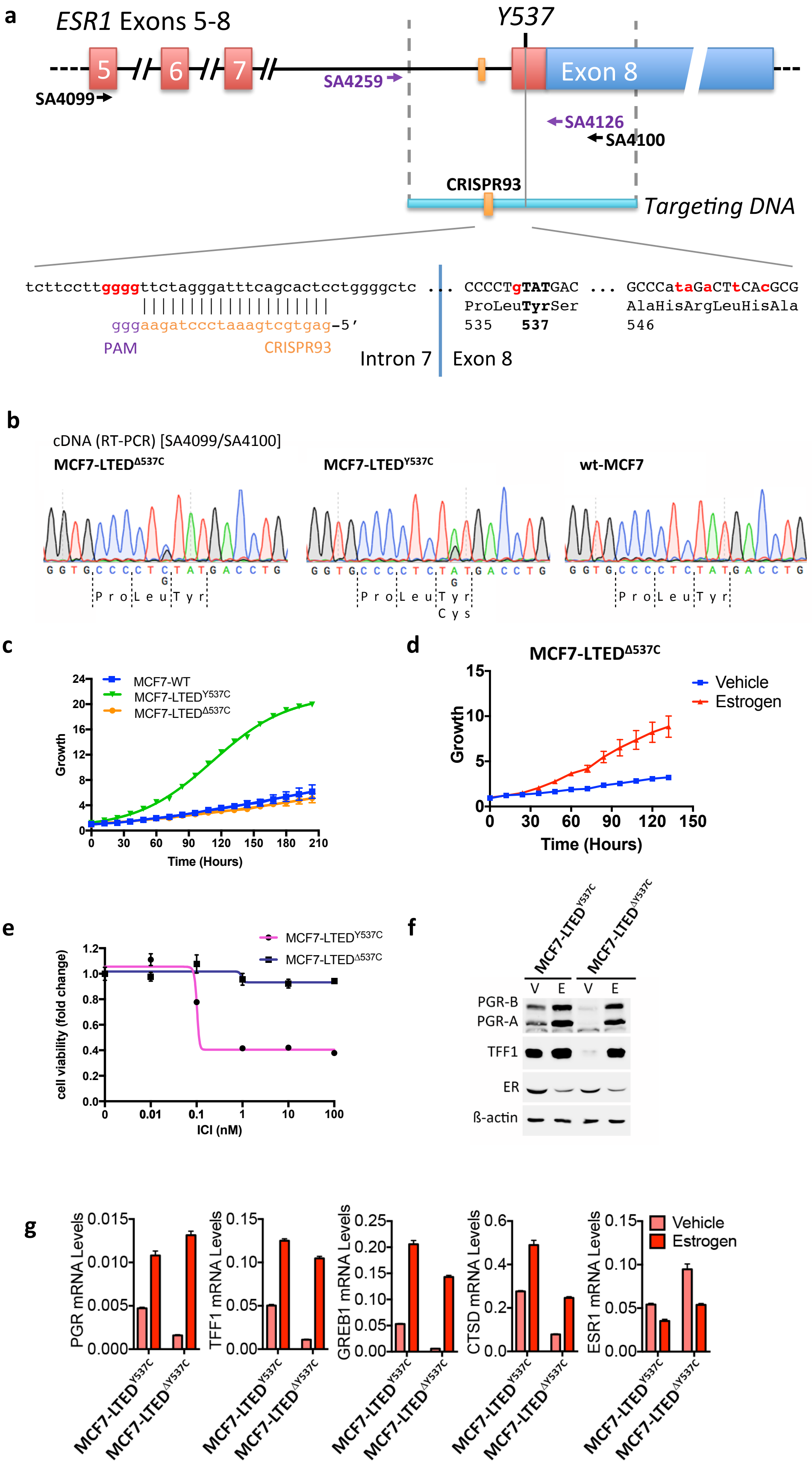

Supplementary Figure 10 CRISPR-Cas9 editing of MCF7-LTED (**a**) Schematic representation of the ESR1 gene showing the positions of PCR primers used in the

study. Also shown is the position of Y537. The targeting DNA comprises a region from within intron 7 to the 3' UTR in exon 8. The position of CRISPR4834039 (aka CRISPR93) is shown as an orange rectangle. The changes introduced into the *ESR1* targeting DNA are highlighted and include replacement of cccc by gggg, to destroy the PAM site in the targeting DNA, a silent change at Leu536 (CTC>CTG) and further silent changes within codons for amino acids 547-550. Clone 3D3A6 was generated following Cas9, CRISPR and target DNA transfection of MCF7-LTED-Y537C cells, as described [23]. **(b)** Sanger sequencing of RT-PCR products using primers in exon 5 (SA4099) and exon 8 (SA4100), shows expression of only $ESR1^{WT}$ and ESR1 in which the silent change in Leu536 has occurred. There is no evidence for expression, at the mRNA level, of the 537C allele. Sequencing of RT-PCR products for $MCF7\text{-}LTED^{Y537C}$ and wt-MCF7 is also shown. (**c**) Temporal growth assay in which the effect of the edited mutation on ligand-independence was assessed. Cells were cultured in DCC medium for 8-days. Data represents growth relative to day 0 (n=3 biological replicates and n=6 technical replicates) (**d**) Temporal growth assay assessing the response of the $MCF7\text{-}LTED^{\Delta537C}$ cells in DCC-medium in the absence (vehicle) or presence of E over a 6-day period. Data represents growth relative to day 0 (n=3 biological replicates and n=6 technical replicates). (**e**) The response of the CRISPR-cas9 edited cell line to escalating concentrations of fulvestrant (ICI) over a 6 day period was assessed in the absence of estrogen showing no further anti-proliferative effect, as expected. In contrast, the $MCF7\text{-}LTED^{Y537C}$ cell line was inhibited confirming its ligand-independent phenotype (n=2 biological replicates and n=8 technical replicates). **(f)** Immunoblotting of cell lysates prepared 24 hours following addition of 10 nM E, shows restoration of estrogen-dependency (**g**) RNA prepared 16 hours following addition of 10 nM estrogen, was used in RT-qPCR assays. Graphs show gene expression relative to GAPDH expression (n=3). Data represents mean ± SEM.

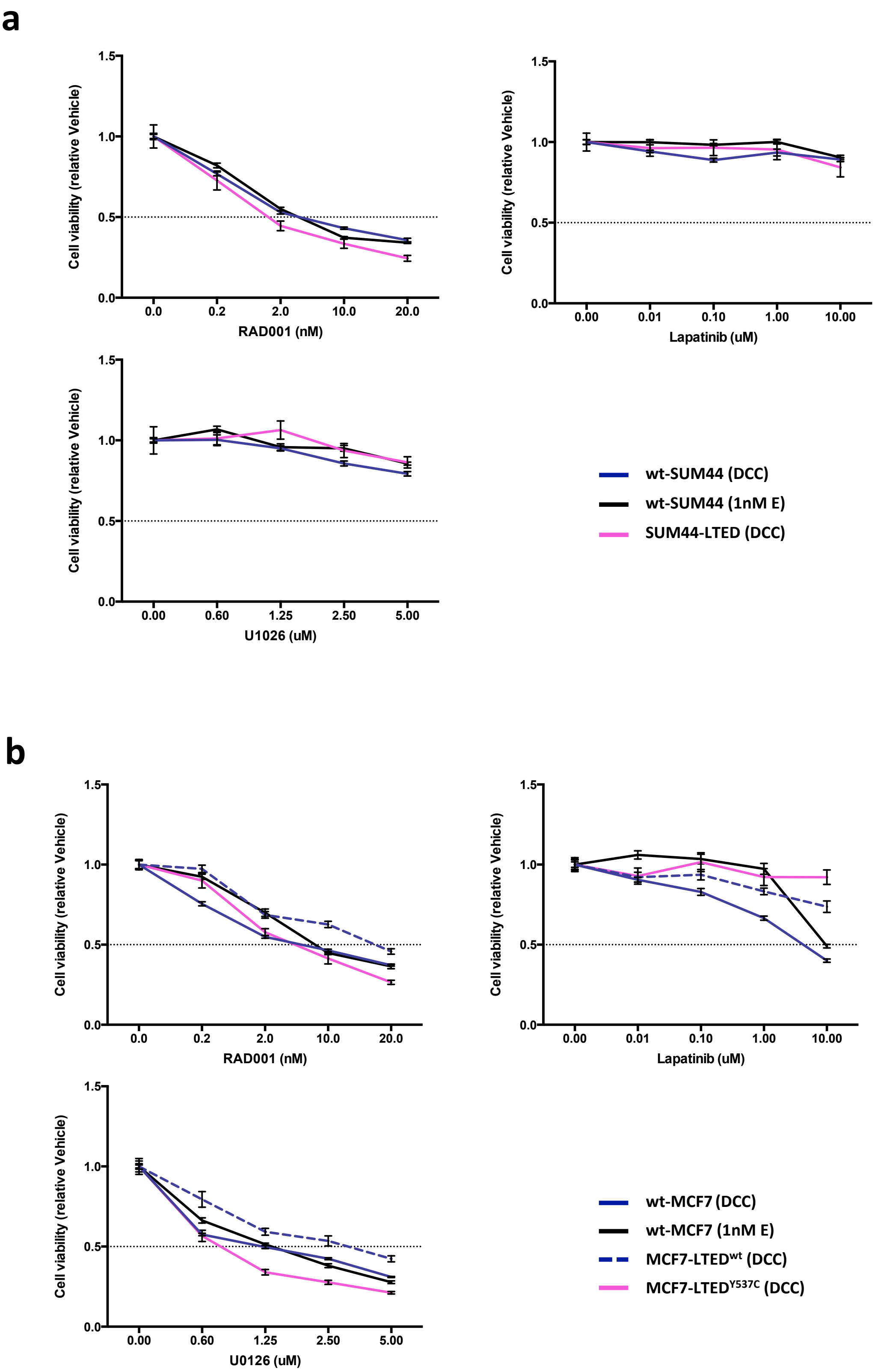

Supplementary Figure 11 The effect of inhibition of ERBB, ERK and MTORC1 pathways in **(a)** SUM44 and **(b)** MCF7 cell lines derivatives. Cells were treated ± estradiol (E) with escalating concentrations of the drugs indicated. Data represents mean ± SEM (n=8)
