# Supplementary material for: Discovery of naturally occurring ESR1 mutations in breast cancer cell lines modelling endocrine resistance
Source: Nat Commun. 2017 Nov 30;8:1865. doi: 10.1038/s41467-017-01864-y (PMC5709387; doi:10.1038/s41467-017-01864-y)
Supplement: Supplementary file 2 — Description of Additional Supplementary Files [file 41467_2017_1864_MOESM2_ESM.pdf]

Description of Additional Supplementary Files

File Name: Supplementary Data 1
Description: Whole exome sequencing showing differences between wt-MCF7, wt-SUM44 and their LTED derivatives.

File Name: Supplementary Data 2
Description: Hallmark pathways identified from the integration of ChIP-seq and RNA-seq data from SUM44-LTED and wt-MCF7$^{Y537S.}$

File Name: Supplementary Data 3
Description: Targeted proteomic analysis for ESR1$^{Y537S.}$
